# Inflammatory and Repair Pathways Induced in Human Bronchoalveolar Lavage Cells with Ozone Inhalation

**DOI:** 10.1371/journal.pone.0127283

**Published:** 2015-06-02

**Authors:** Pascale Leroy, Andrea Tham, Hofer Wong, Rachel Tenney, Chun Chen, Rachel Stiner, John R. Balmes, Agnès C. Paquet, Mehrdad Arjomandi

**Affiliations:** 1 San Francisco Veterans Affairs Medical Center, San Francisco, California, United States of America; 2 Human Exposure Laboratory, Division of Occupational and Environmental Medicine, University of California San Francisco, San Francisco, California, United States of America; 3 Division of Pulmonary, Critical Care, Allergy and Immunology, and Sleep Medicine, University of California San Francisco, San Francisco, California, United States of America; 4 Lung Biology Center, Department of Medicine; University of California San Francisco, San Francisco, California, United States of America; 5 Division of Environmental Health Sciences, School of Public Health, University of California, Berkeley, California, United States of America; 6 Centre National de la Recherche Scientifique, Institut de Pharmacologie Moléculaire et Cellulaire, Valbonne, France; University of Kentucky, UNITED STATES

## Abstract

**Background:**

Inhalation of ambient levels of ozone causes airway inflammation and epithelial injury.

**Methods:**

To examine the responses of airway cells to ozone-induced oxidative injury, 19 subjects (7 with asthma) were exposed to clean air (0ppb), medium (100ppb), and high (200ppb) ambient levels of ozone for 4h on three separate occasions in a climate-controlled chamber followed by bronchoscopy with bronchoalveolar lavage (BAL) 24h later. BAL cell mRNA expression was examined using Affymetrix GeneChip Microarray. The role of a differentially expressed gene (DEG) in epithelial injury was evaluated in an *in vitro* model of injury [16HBE14o- cell line scratch assay].

**Results:**

Ozone exposure caused a dose-dependent up-regulation of several biologic pathways involved in inflammation and repair including chemokine and cytokine secretion, activity, and receptor binding; metalloproteinase and endopeptidase activity; adhesion, locomotion, and migration; and cell growth and tumorigenesis regulation. Asthmatic subjects had 1.7- to 3.8-fold higher expression of many DEGs suggestive of increased proinflammatory and matrix degradation and remodeling signals. The most highly up-regulated gene was osteopontin, the protein level of which in BAL fluid increased in a dose-dependent manner after ozone exposure. Asthmatic subjects had a disproportionate increase in non-polymerized osteopontin with increasing exposure to ozone. Treatment with polymeric, but not monomeric, osteopontin enhanced the migration of epithelial cells and wound closure in an α9β1 integrin-dependent manner.

**Conclusions:**

Expression profiling of BAL cells after ozone exposure reveals potential regulatory genes and pathways activated by oxidative stress. One DEG, osteopontin, promotes epithelial wound healing in an *in vitro* model of injury.

## Introduction

Ozone, a potent oxidant gas, is a major component of air pollution to which millions of people are regularly exposed. Upon inhalation, ozone interacts with airway lining fluid in the lungs to produce ozonation products and reactive oxygen species (ROS), which result in oxidative stress. Animal and human exposure studies have documented that ozone-induced oxidative stress causes a multitude of events including an immediate influx of granulocytic inflammatory cells, recruitment of monocytic cells, activation of alveolar macrophages, and toxicity and injury to airway epithelial cells as well as lung function decrements [1–8].

Although the cascade of mechanisms by which ozone inhalation produces its airway toxicity has been extensively studied, the mechanisms by which ozone-induced oxidative stress and injury is resolved are not established. Since oxidative injury is a common etiology in pathogenesis of many respiratory diseases, identifying the biological pathways that are responsible for attenuation of inflammation and resolution of injury in lungs after ozone-inhalation could have important implications. While the function of granulocytic inflammation associated with ozone-induced injury has been studied [9, 10], the role of other airway inflammatory cells in this process is less known. Previous studies have shown that the ozone-induced granulocytic inflammation peaks at 6 hours, persists to about 18 to 20 hours, and then attenuates at 24 hours [11]. Other studies have shown that repeated stress and injury by inhalation of ozone causes an increase in recruitment of macrophages into airways [12, 13]. Macrophages constitute the majority of airway immune cells within the lumen of airways, and, at least in other tissues, are known to play a role in repair processes initially through activation of inflammatory processes to remove injured cells, and then through suppression of inflammation, clearance of cellular debris, and assistance with extracellular matrix repair [14–16]. Thus, it is plausible that airway immune cells may contribute to resolution of ozone-induced oxidative stress and injury.

The goal of this study was to identify the biological processes involved in ozone-induced oxidative stress and injury, particularly with respect to resolution of inflammation and promotion of tissue repair. To do this, we examined the gene expression of bronchoalveolar lavage cells after exposure to clean air and medium and high ambient levels of ozone. We then examined the role of one of the highly differentially expressed genes, secreted phosphoprotein 1 (SPP1, the gene for osteopontin), with demonstrated functions in adhesion, migration, and repair processes in skin and bone tissues, in airway epithelium wound repair using an *in vitro* model of injury and repair.

## Methods

### Ethics Statement

The University of California San Francisco (UCSF) Institutional Review Board (IRB) and the Committee on Human Research approved this study. Written IRB-approved informed consent was obtained from all study participants. All subjects received financial compensation for their participation.

### Study Design

This study had a repeated measure design in which subjects were exposed to either 0 ppb (filtered air with no ozone added), 100 ppb (medium dose), or 200 ppb (high dose) ozone for 4 hours in a climate-controlled chamber followed by bronchoscopy with bronchoalveolar lavage (BAL) approximately 24 hours later (20 hours after the end of exposure). The 100ppb and 200ppb concentrations, which represent medium and high ozone ambient levels in some metropolitan areas in the US and around the world, were chosen based on the assumption that the threshold for the oxidative stress effect of ozone on airway cells is below these levels, as suggested by previous studies [17, 18].

Each subject underwent all three exposure with a minimum of 3 weeks in between exposure sessions to allow for recovery from any inflammation or injury sustained during the prior session. Previous studies have shown this “wash-out” period to be adequate for controlled ozone exposure studies [19, 20]. The order of exposures was counterbalanced and randomized. The study subjects and all but one of the study personnel, who performed the exposure experiment, were blinded to the type and order of exposures.

Additional methods including the details of spiromtery, preparation of polymeric osteopontin, and immunoblot assays are available in Supplemental S1 Methods.

### Subjects

Twenty-six subjects were initially recruited. Three subjects refused bronchoscopy and four subjects withdrew from the study after the first exposure and bronchoscopy. Nineteen subjects completed all exposures and bronchoscopies. Due to drop out of subjects, the order of exposure assignments in those who completed the study was not entirely counter-balanced, and there was an uneven distribution of subjects who completed the different orders of exposure assignments (S1 Table). Nevertheless, the differences between the number of subjects in exposure-order assignments were not statistically significantly different (chi-squared p-value = 0.09).

The inclusion criteria included: (1) age between 18 to 50 years; (2) ability to perform moderate-intensity exercise; (3) healthy with no history of cardiovascular, hematologic, or pulmonary diseases other than mild asthma; (4) no history of acute infection within the past 6 weeks prior to start of the study; (5) non-smoker as defined by having a history of <½ pack-year/lifetime tobacco use and no history of any tobacco use in the past 6 months; and (6) no history of illicit drug use. The criteria for mild asthma diagnosis included a self-report of physician-diagnosed asthma, airway hyperresponsiveness to inhaled methacholine [provocative concentration of methacholine resulting in a 20% decrease in FEV_1_ compared to baseline (PC_20_) ≤8.0 mg/ml] verified in our laboratory according to a protocol based on the American Thoracic Society guidelines [21], a pre-bronchodilator FEV_1_ of ≤70% of the normal predicted value, no daily asthma symptoms, and less than 3 to 4 nighttime symptoms per month [22, 23]. Asthmatic subjects were asked to, and had to be able to stop their asthma and allergy medications in a sequential manner based on the duration of action of each medication (inhaled corticosteroids for 2 weeks, anti-histamines and leukotriene inhibitors for 3 days, long-acting bronchodilators for 2 days, and short-acting bronchodilators for 8h).

### Climate-Controlled Chamber and Atmospheric Monitoring

The experiment took place in a ventilated, climate-controlled chamber at 50% relative humidity and 18°C (the temperature in the chamber was adjusted for subjects’ comfort based on previous experience). The chamber is a stainless steel-and-glass room of 2.5×2.5×2.4 cubic meters (Model W00327-3R; Nor-Lake, Hudson, WI) that was custom-built and designed to maintain temperature and relative humidity within 2°C and 4% from the set points, respectively (WebCtrl Software; Automated Logic Corporation, Kennesaw, GA). Temperature and relative humidity were recorded every 30 seconds and displayed in real-time (LabView 6.1; National Instruments, Austin, TX).

### Exposure Sessions and Bronchoscopy and Lavage Procedures

Each exposure session was 4 hours long, with subjects exercising for the first 30 minutes and then resting for the following 30 minutes of each hour in the climate-controlled chamber. The exercise consisted of running on a treadmill or pedaling a cycle ergometer. Exercise intensity was adjusted for each subject to achieve a target expired minute ventilation (V_E_) of 20 L/min/m^2^ body surface area. Bronchoscopies were performed 20±1h after the end of each exposure according to our laboratory’s established bronchoscopy and BAL procedures [24]. Briefly, the bronchoscope was advanced into the right middle lobe bronchus and after obtaining a “wedge”, BAL was performed with instillation of two 50-ml aliquots of 0.9% sterile saline warmed to 37°C and then application of gentle suction. The BAL was collected in a polyethylene tube and placed on ice transiently during transport to laboratory. A small aliquot (1 ml) of the BAL was separated for performance of cell count, and the remainder was immediately fractionated into cells and fluid using centrifugation at 180 g for 15 minutes at 4° C. The supernatant (BAL fluid) was separated and frozen at -80° C. A portion of the BAL cell pellet (BAL cells) was immediately placed in RLT buffer (Qiagen, Venlo, Limburg) and then stored at -80° C for later RNA work.

### RNA Extraction from BAL Cells

BAL cells in RLT buffer were transported on dry ice to Expression Analysis, Inc. for RNA extraction and gene expression analysis. RNA was extracted from BAL cells stored in RLT buffer (Qiagen) using the RNeasy kit (Qiagen), and concentration and quality were assessed using the Bioanalyzer 2100 (Agilent Technologies, Santa Clara, CA).

### Gene Expression Microarray Assay

Microarray data were collected at Expression Analysis, Inc. (www.expressionanalysis.com; Durham, NC). Biotin-labeled target for the microarray experiment was prepared using 100 ng of total RNA and cDNA was synthesized using the GeneChip WT (Whole Transcript) Sense Target Labeling and Control Reagents kit as described by the manufacturer (Affymetrix, Santa Clara, CA). The sense cDNA was then fragmented by UDG (uracil DNA glycosylase) and APE 1 (apurinic/apyrimidic endonuclease 1) and biotin-labeled with TdT (terminal deoxynucleotidyl transferase) using the GeneChip WT Terminal labelling kit (Affymetrix). Hybridization was performed using 5 micrograms of biotinylated target, which was incubated with the GeneChip Human Gene 1.0 ST array (Affymetrix) at 45°C for 16–20 hours. Each sample was hybridized with a dedicated array. Following hybridization, non-specifically bound material was removed by washing and detection of specifically bound target was performed using the GeneChip Hybridization, Wash and Stain kit, and the GeneChip Fluidics Station 450 (Affymetrix). The arrays were scanned using the GeneChip Scanner 3000 7G (Affymetrix) and raw data were extracted from the scanned images and analyzed with the Affymetrix GeneChip Command Console Software (AGCC, Affymetrix).

Microarray data have been deposited in the NCBI Gene Expression Omnibus (Accession Number GSE58682).

### Bronchial Epithelial Wound Closure Assay

Human bronchial epithelial cells (16HBE14o-) were developed and generously provided by Dr. Deiter Gruenert from the University of California San Francisco [25]. The cells were seeded and grown to confluence on 24-well trans-well membrane plates in FBS-supplemented with MEM media. Once confluent, the cells were treated with PBS (negative control), monomeric rOPN (50 ng/ml), polymeric rOPN (50 ng/ml), or TG2 (20 ng/ml; a very low concentration that was used for polymerization of monomeric OPN) for 72 h. After this period, a scratch in the epithelial cell layer was then made with a plastic 200 μl pipet tip[26]. The cells were immediately rinsed to remove the cell debris and then incubated with PBS (control), monomeric rOPN (50 ng/ml), polymeric rOPN (50 ng/ml), or TG2 (20 ng/ml). To determine the potential role of α_9_β_1_ integrin, the cells were also treated with PBS (negative control), anti-human OPN antibodies (50 ng/ml) (R&D Systems), and anti-human α_9_ antibody (50 ng/ml) (Y9A2) generously provided by Dr. Dean Sheppard from the University of California San Francisco Lung Biology Center) after the scratch was made in separate set of experiments. Scratch closure was observed and cells were fixed with PFA 4% after 14 h. The 14-hour time point was chosen to allow for partial closure of the scratch in the epithelial cell layer so that the effect of various treatments could be assessed. The cells were labeled with DAPI and pictures were taken for quantification using Image J software. Quantification was comprised of measurement of the wound area and length by a blinded observer. The final wound area used in analysis was normalized for the wound length to obtain average scratch width.

### Transepithelial Electrical Resistance

To verify epithelial layer formation and polarization of the 16HBE14o- cells, we measured the transepithelial electrical resistance (TEER) of the cell layers on transwell membrane plates before the scratch was made. TEER was measured using an ohmmeter (EVOM, World Precision Instruments, Sarasota, FL) after pre-treatment with PBS (negative control), monomeric rOPN (50 ng/ml), polymeric rOPN (50 ng/ml), or TG2 (20 ng/ml) and immediately before the scratch was made.

The TEER values ranged from 820 to 1953 ohms/cm^2^ [(mean ± SD) 1313 ± 276 ohms/cm^2^] with >90% of measurements above the 1000 ohms/cm^2^ reported level for well-formed epithelial layers. Pre-treatment with monomeric OPN (mOPN) or polymeric OPN (pOPN) did not significantly affect TEER values before scratch compared to treatment with PBS (mean ± SD of PBS: 1188 ±241.7; mOPN: 1359 ± 276; and pOPN; 1337±253 ohms/cm^2^).

### Flow Cytometry of 16HBE14o- Cells

16HBE14o- cells were grown to confluence and were then allowed to grow for an additional three days. Cells were detached using trypsin (Lonza, Basel, Switzerland), labeled with the corresponding antibodies (anti-α9 antibody Y9A2, anti-β1 antibody P5D2, anti-β5 antibody ALULA, and anti-β6 antibody 3G9) generously provided by Dr. Dean Sheppard, and analyzed using flow cytometry [27].

### Immunoblot of α9 Integrin in 16HBE14o- Cells

16HBE14o- cell lysates at baseline and after each treatment were prepared using RIPA lysis buffer as per manufacturer protocol (Millipore). The concentration of α9 integrin protein in the cell lysate was then measured by western blot assays as described previously [28] using anti-human α9 integrin antibody Y9A2, and quantified using Image J software as previously described [28].

### Sample Size and Power Calculations

Human inhalational exposure studies have reported significant changes in bronchoalveolar lavage and blood inflammatory biomarkers with sample sizes from 15 to 30 subjects [24, 29–31]. From previous Taqman-based measurements of interleukin-6 (IL-6) RNA levels (unpublished data from our laboratory), we have observed differences (in log copy number) of 1.56 with associated standard deviation (SD) of 1.80. Given the above, sample sizes of 20 and 25 provided statistical power of 78% and 86%, respectively, to observe such a change at two-sided significance level of 0.05. Our final sample size of 19 subjects provided a power of 76% at two-sided significance level of 0.05.

While some approaches have been proposed for performing power/sample size determination in the context of microarray investigation of differential expression [32, 33], these required unavailable inputs and were problematic for our purposes. Differential expression has been established, with stringent adjustment for multiplicity, in human microarray studies using similar sample sizes as in our study [34]. Moreover, reduced variability due to the repeated measure design of our study further increased its statistical power. In addition, in a previous separate preliminary gene expression study, we examined the differentially expressed genes (DEGs) of BAL cells in 3 subjects after exposure to 0 and 200 ppb ozone [35]. The candidate DEGs were selected based on FDR of <0.05 level and log-odds ratio of being differentially expressed to not being differentially expressed (B) >0. Overall, 121 genes were identified as DEGs in this preliminary study (S2 Table). A selection of these DEGs with a B-value >2 that are known to be involved in leukocyte migration, immune response, inflammation, extracellular region, and tissue repair and remodeling (based on analysis using DAVID) were chosen *a priori* as candidate DEGs for re-examination in this larger sample size of 19 subjects.

### Data Management and Statistical Analysis

All data were entered into a database (Microsoft Excel 2003; Microsoft; Redmond, WA) and then analyzed using STATA statistics software (STATA IE, version 12.0; StataCorp; College Station, TX). Each subject served as his or her own control. Data are presented as mean ± SD or as median [interquartile range]. A p-value of <0.05 was considered to be statistically significant in all analyses. All data distributions were examined before analysis. Student's *t*-test or Wilcoxon signed-rank test were used for initial pair-wise comparisons based on the distribution of data. The change in a particular variable over the course of each exposure was calculated linearly using the 0-h value as the baseline. To determine the presence of a dose-dependent response effect, normality of the data distribution was examined, concentrations were log-transformed to meet the normality assumption if needed, and a population-averaged generalized linear model was calculated.

### Statistical Methods for Microarray Data Analysis

The precise methodology used in the analysis of gene expression to summarize and normalize the data is described at the Expression Analysis Inc. technical note website (http://www.expressionanalysis.com/docs/PADE_Tech_Note.pdf). Briefly, the analysis was conducted on normalized expression values that were individually transformed using the base 2 logarithm of the relevant expression index (e.g., MAS5). A floor of 1.0 was used to avoid negative transformed values [log2(max(1.0,Signal))]. After computing the mean for each group using the log transformed signal values, the mean was then converted back to the original probeset signal units. The averaging on the logarithm scale and then inverse transformation (also known as the geometric averaging) provides a more robust estimate of overall expression that is less impacted by outliers or skewed expression levels relative to a simple arithmetic average of the raw expression values from each array. Raw fold change was calculated as the simple ratio of overall expression values from the two groups. The higher overall expression was divided by the lower overall expression. If the baseline group expression was higher, then fold change was designated to have a negative value.

Tests for linear differential expression were performed using LIMMA[36], and tests for two-group comparisons were performed using permutation analysis for differential expression (PADE). Specifically, for linear analysis, three models were used. The first linear model was fit with terms for dose [0ppb, 100ppb, and 200ppb] and subject. Contrasts were constructed to test for differential expression at each dose (100 and 200 ppb) relative to 0 ppb. The log_2_ fold change and p-value was calculated for each contrast. A second model was fit with terms for presence of asthma (binary) and subject, and a third model contained terms for presence of lung function response to ozone defined as >5% decline in FEV_1_ across exposure (binary) and subject. Effects and significance (p-values) were recorded for the asthma and FEV_1_ variables in these models. The Benjamini and Hochberg transformation of p-values was performed to estimate the q-value for each gene and allow control of the false discovery rate (FDR) in all three comparisons[37]. FDR is defined as the expected proportion of false positive findings among those differentially expressed, and the q-value is defined as the individual measure of significance for each probe set in terms of the FDR. Prior to visualization, the intra-subject expression values were median centered for each gene.

Although linear regression was specified *a priori* to be the analytical plan for analysis of gene expression data, *post-hoc* two-group comparisons were also conducted for filtered air (FA)-100 ppb, 100 ppb-200 ppb, and FA-200 ppb condition sets to examine changes in expression of genes between baseline and experimental groups. Significance of differential gene expression was based on a calculated permutation analysis for differential expression (PADE) delta threshold value specific to each two-group comparison [38]. Cluster analysis and heatmaps of differentially expressed genes were created using Pearson correlation as the distance measure and complete linkage clustering.

To examine the effect of clinical covariates (sex, asthma, atopy or allergic status, lung function response, neutrophilic response, and eosinophilic response) on ozone-induced gene expression of BAL cells, both (1) non-hierarchical cluster analysis (bottom-up approach); and (2) analysis using stratification of subjects by covariates (top-down approach) were performed. In the first approach, DEGs based on the clinical covariates were determined by cluster analysis at baseline (0 ppb) exposure using the hclust function of R language[39], and then the effect of ozone on these genes at 100 and 200 ppb exposures was examined. In the second approach, subjects were stratified by clinical covariates, and then differential gene expression in response to ozone exposure was examined in each stratum. Atopy or allergic status was defined by a positive response to allergy skin prick tests. Lung function response was defined as >5% decline in FEV_1_ from before to immediately after 200 ppb ozone exposure. Neutrophilic and eosinophilic responses were defined as >50% increase in neutrophil or eosinophil concentrations in BAL after 200 ppb ozone exposure compared to 0 ppb exposure, respectively.

DAVID (www.david.abcc.ncifcrf.gov, NIAID, NIH, Frederick, MD) was used to explore enriched ontologies associated with the differentially expressed genes in the Gene Ontology Biological Process database [40, 41]. Ingenuity Pathway Analysis (IPA) was used to perform Gene Set Analysis (GSA) for identification of differential expression of Biocarta, Gene Ontology, and KEGG pathways or gene lists using dose as a three class categorical variable [42]. Benjamini and Hochberg transformation of p-values was performed to control the FDR for gene set association. Processes with FDR threshold of <0.25 were considered to be significant. Ingenuity iReport (Ingenuity Systems, www.ingenuity.com; Redwood City, CA) was also used to identify diseases, pathways and processes known to be associated with the differentially expressed genes. As it only generates uncorrected p-values, FDR was not reported for Ingenuity iReport analysis.

## Results

### Subject Characteristics, Exposure Conditions, and BAL Inflammatory Changes

**Fig 1:**
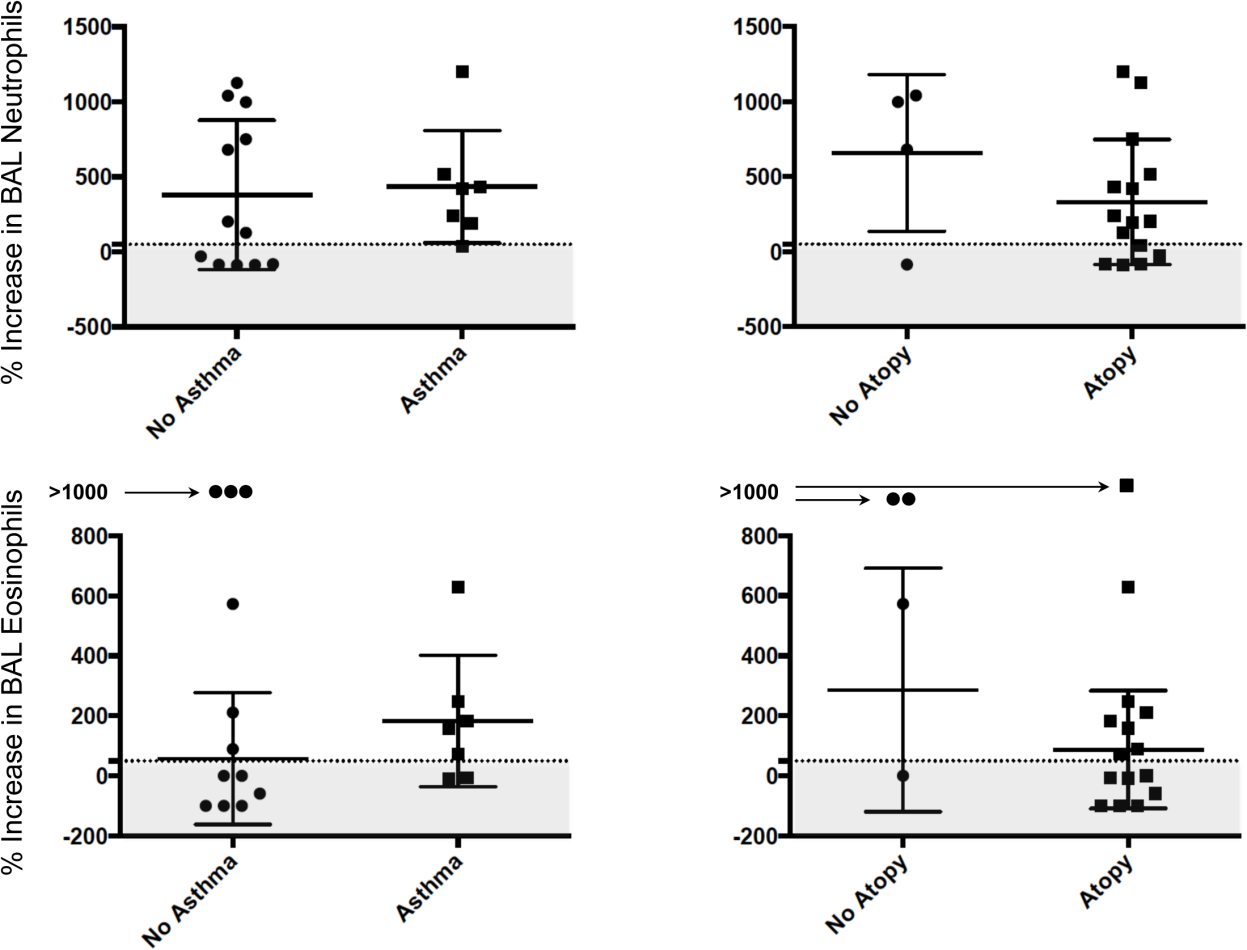
Ozone-induced Neutrophilia and Eosinophilia in Bronchoalveolar Lavage. The changes in bronchoalveolar lavage (BAL) neutrophils and eosinophils from 0 to 200 ppb ozone exposure were not significantly different between the subjects with or without asthma or atopy. The horizontal dashed lines represent the 50% increase in count of neutrophils or eosinophils from 0 to 200 ppb ozone exposure. The shaded gray areas represent the subjects categorized as those without an ozone-induced neutrophilic or eosinophilic response (non-responders).

The characteristics of the 19 participating subjects are shown in Table 1. Seven out of the 19 subjects had mild asthma, and 15 out of 19 subjects (including all asthmatic subjects) had atopy. Compared to the subjects without asthma, the subjects with asthma had slightly lower FEV_1_ and FEV_1_ to FVC ratios. Minute ventilation was similar between exposures for each subject (S3 Table) and between subjects with and without asthma across different exposures (Table 1). The overall average temperature and relative humidity in the climate-controlled chamber were 16.4±1.9°C and 53.3±10.7%, respectively. The concentrations of the cells recovered in BAL including the bronchoalveolar lavage inflammatory cells, are shown in Table 2. The concentrations of both BAL neutrophils and eosinophils increased linearly with ozone exposure, but there was no significant change in the concentrations of other BAL cells across exposures. The changes in BAL neutrophils or eosinophils across exposures were not significantly different between the subjects with or without asthma or atopy. There was no significant difference in number of subjects categorized as neutrophilic or eosinophilic responders (defined by >50% increase in count at 200 ppb exposure) between those with or without asthma or atopy (Fig 1).

**Table 1:** Bronchoscopy Subjects Characteristics.

Subject Characteristics	All Subjects	Non-asthmatic	Asthmatic	p-value
Number	19	12	7	-
Age (years)	32.5±7.6	31.8±6.0	33.7±10.1	0.59
Sex (female) (N [%])	9 [47%]	5 [42%]	4 [57%]	0.54
Height (cm)	171±10	168±11	175±8	0.14
BMI (kg/m^2^)	25.4±5.0	24.8±3.2	26.5±7.3	0.49
FEV_1_ (L)	3.5±0.8	3.8±0.8	3.1±0.6	0.05
FEV_1_ (% predicted)	97.9±15.1	104.3±14.4	87.1±9.3	**0.01**
FVC (L)	4.6±1.0	4.6±1.0	4.6±1.1	0.94
FVC (% predicted)	102.2±12.7	104.9±13.2	97.4±11.3	0.22
FEV_1_/FVC ratio	77.5±10.0	82.5±4.4	69.0±11.3	**0.001**
BSA (m^2^)	1.85±0.23	1.79±0.21	1.95±0.25	0.15
Atopy (N [%])	15 [79%]	8 [67%]	7 [100%]	0.09
V_E_ (L/min/m^2^ BSA) with 0 ppb O_3_	22.1±2.2	22.6±2.0	21.2±2.3	0.18
V_E_ (L/min/m^2^ BSA) with 100 ppb O_3_	22.2±3.5	21.8±3.3	22.9±3.9	0.51
V_E_ (L/min/m^2^ BSA) with 200 ppb O_3_	22.0±3.4	22.7±2.8	20.8±4.1	0.23

Data presented as mean±SD. BMI: body mass index; FEV_1_: forced expiratory volume in 1 second; FVC: forced vital capacity; V_E_: average minute ventilation during exercise; BSA: body surface area; O_3_: ozone. P-value is for comparison between asthmatic and non-asthmatic subjects.

**Table 2:** Ozone-induced cellular inflammation in airways.

Cells Recovered in BAL		0 ppb O_3_	100 ppb O_3_	200 ppb O_3_	PE±SEM (x10^4^ cells/ml per 100 ppb increase in O_3_)	p-value
Leukocytes (x10^4^ cells/ml)		20.6±11.5	21.5±10.6	22.6±8.8	0.97±0.87	0.26
	Macrophages (x10^4^ cells/ml)	17.3±10.0	17.6±10.0	18.1±6.9	0.40±0.83	0.63
	Neutrophils (x10^4^ cells/ml)	0.55±0.56	0.94±0.91	1.51±1.79	**0.48±0.20**	**0.01**
	Lymphocytes (x10^4^ cells/ml)	2.8±2.0	2.7±1.5	2.6±2.1	-0.08±0.24	0.72
	Eosinophils (x10^4^ cells/ml)	0.14±0.17	0.23±0.26	0.37±0.49	**0.11±0.05**	**0.02**
Epithelial Cells (x10^4^ cells/ml)		0.98±1.21	0.74±0.93	0.88±1.06	-0.05±0.16	0.77
Squamous Cells (x10^4^ cells/ml)		0.33±0.26	0.18±0.18	0.26±0.28	-0.03±0.04	0.43
RBC (x10^4^ cells/ml)		10.3±10.0	8.0±8.2	19.7±39.8	4.70±3.83	0.22

Data presented as mean ± standard deviation (SD) and parameter estimate ± standard error of mean (PE±SEM) for linear regression models. N = 19; p-values are from the regression model of each BAL inflammatory cell against level of ozone exposure. O_3_: ozone; RBC: red blood cells. Significant comparisons are shown in bold.

### Ozone Exposure Caused a Dose-dependent Up-regulation of Several Genes Involved in Injury, Inflammation, and Repair Processes

**Fig 2:**
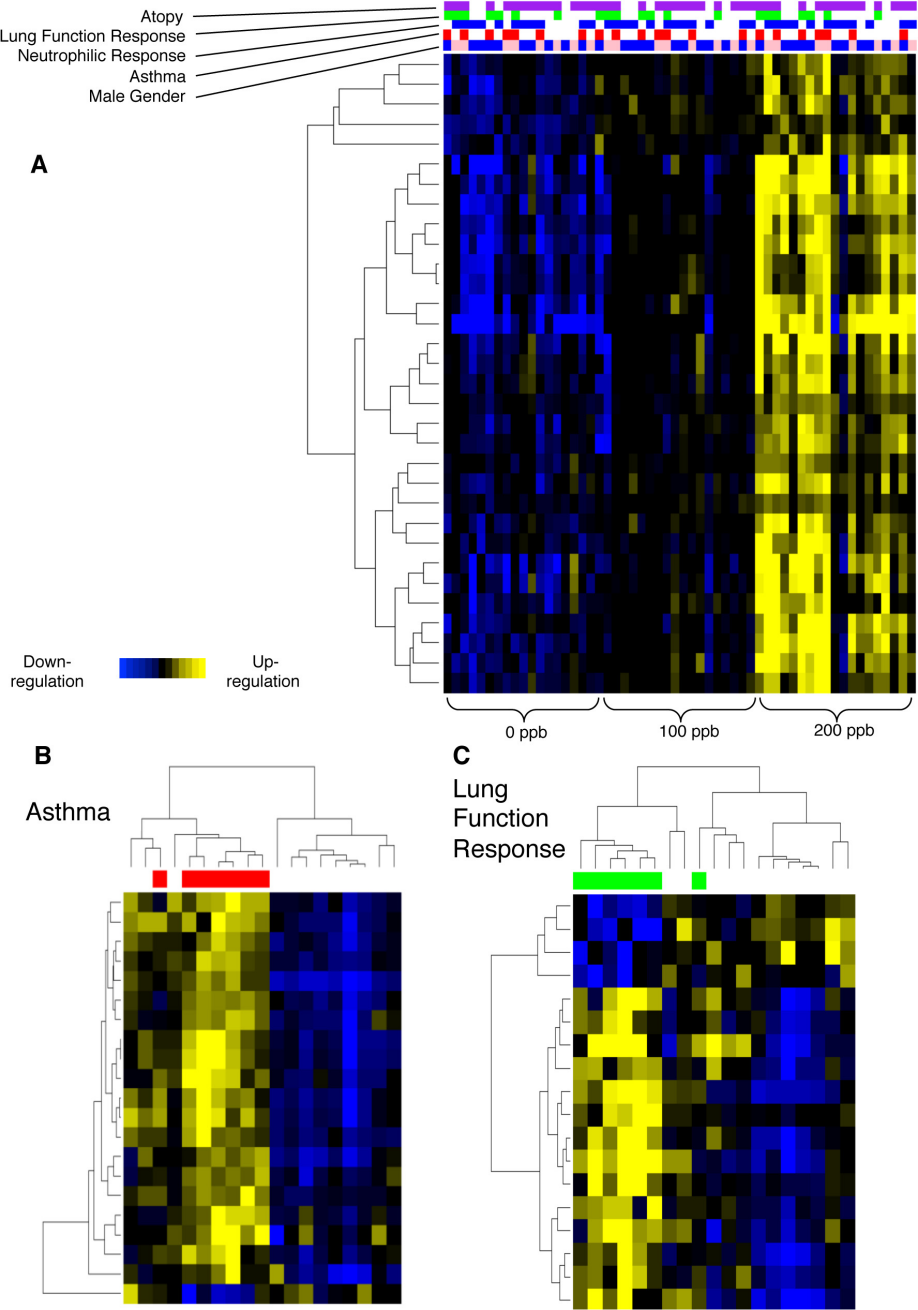
Heatmap of BAL Cell Gene Expression After Ozone Exposure. **A**. Ozone exposure had a consistent effect on the gene expression of human BAL cells with up-regulation of a group of genes in a dose-dependent manner from 0 to 100 to 200 ppb. The subjects are presented on the horizontal axis in the same order in each level of exposure. Covariates of atopy, lung function response, neutrophilic response, asthma, and gender are marked above the heatmap, with color indicating presence of covariates (for gender, blue = male; pink = female; for lung function and neutrophilic response, green and blue = no response; see Methods for details). **B & C**. Cluster analysis identified a relatively distinct classification of subjects with and without asthma or those with and without lung function response to 200 ppb ozone.

Analysis of gene expression data using linear regression showed up-regulation of 30 genes in the BAL cell samples with exposure to increasing levels of ozone (q-value <0.05) (Table 3). Linear regression analysis did not show any down-regulated genes. Using a q-value between 0.05 and 0.10, an additional 13 genes were also found to be up-regulated. Fig 2 part A shows a heat map of the differentially expressed genes (DEGs) for each subject with exposure to increasing levels of ozone.

**Table 3:** Differentially Expressed Genes with Increasing Levels of Ozone Exposure.

	Regression Analysis			PADE Pair-wise Analyses		
Gene Descriptor	100 ppb O_3_ (fold change)	200 ppb O_3_ (fold change)	Regression q-value	0 vs. 100 ppb O_3_ (fold change)	100 vs. 200 ppb O_3_ (fold change)	0 vs. 200 ppb O_3_ (fold change)
**PADE and Regression Significant**						
**SPP1**	2.12	5.96	<0.001	2.14	2.68	5.73
**CCL2**	1.21	2.82	0.03	1.26	2.31	2.9
**IL8RA**	1.24	2.48	0.01	1.34	2.21	2.96
**S100A12**	1.20	2.41	<0.001	1.25	2.22	2.78
**PLXNC1**	1.17	2.24	<0.001	1.19	1.98	2.36
CD1E	1.31	2.17	<0.001	1.31	1.7	2.23
MERTK	1.13	2.10	<0.001	1.17	1.93	2.26
**CD1C**	1.24	2.08	<0.001	1.24	1.7	2.11
**IL1R2**	1.22	2.07	<0.001	1.31	1.77	2.33
RASSF2	1.11	1.94	0.02	1.09	1.85	2.01
**KCNJ15**	1.17	1.89	0.01	1.28	1.65	2.12
GPR84	1.10	1.63	0.01	1.16	1.56	1.81
ACPP	1.09	1.63	<0.001	1.06	1.61	1.7
ST8SIA4	1.00	1.53	0.03	-1.01	1.55	1.54
MEF2C	-1.01	1.52	0.02	-1.03	1.59	1.54
**Regression Significant Only**						
**PLA2G7**	1.43	2.65	<0.001	1.48	1.75	2.59
LAMP3	1.28	1.82	<0.001	1.31	1.4	1.84
CCL22	1.24	1.81	<0.001	1.24	1.52	1.89
PI3	1.21	1.70	0.04	1.26	1.52	1.91
CX3CR1	1.18	1.69	<0.001	1.18	1.53	1.8
MMP9	1.31	1.68	0.01	1.36	1.33	1.82
PRKCB	1.09	1.60	0.01	1.1	1.5	1.66
CHST15	1.14	1.54	0.04	1.17	1.43	1.67
ETV5	1.22	1.51	0.02	1.25	1.24	1.55
MGAM	1.16	1.45	0.02	1.18	1.26	1.49
MMP8	1.20	1.45	0.03	1.25	1.2	1.5
HSD11B1	1.32	1.42	<0.001	1.35	1.08	1.46
ANKH	1.03	1.29	0.01	1.01	1.3	1.32
SIGLEC10	1.06	1.29	0.01	1.06	1.25	1.33
SEMA4A	1.10	1.24	0.02	1.09	1.18	1.29
PADE Significant Only						
STEAP4	1.18	2.24	1.00	1.40	1.89	2.65
GPR183	1.19	1.95	0.07	1.18	1.69	2.00
ANKRD22	1.14	1.82	0.17	1.12	1.75	1.96
CCR2	1.04	1.73	0.34	-1.01	1.79	1.78
SERPINB9	1.08	1.72	0.11	1.05	1.67	1.75
C4orf18	1.06	1.64	0.14	1.01	1.7	1.73
CD62L	-1.01	1.59	1.00	1.00	1.73	1.72
SELL	1.00	1.59	1.00	1.01	1.7	1.72
CORO1A	1.00	1.54	1.00	-1.05	1.69	1.61
CLEC5A	1.02	1.51	1.00	-1.02	1.6	1.56
SLC25A37	-1.01	1.49	0.79	1.03	1.6	1.65
**SULF2**	1.13	1.48	0.06	1.11	1.42	1.57
FAM65B	-1.02	1.39	1.00	-1.01	1.53	1.51
LILRA1	1.02	1.38	0.07	-1.01	1.47	1.46
IFITM2	-1.04	1.21	1.00	-1.04	1.37	1.33

The significant differentially expressed genes (DEGs) from both regression and PADE pair-wise analyses are listed with fold changes and q-values from regression analysis, as well as PADE delta values and fold changes by PADE analysis. A fold change of 1.00 would reflect no change. The DEGs are separated into three sections: genes found to be differentially expressed by both linear regression and PADE analyses; genes found to be differentially expressed only by linear regression analysis (q-value), and genes found to be differentially expressed only by PADE delta analysis. For PADE pairwise analyses, only DEGs that were consistently regulated across all three pairwise comparisons with PADE delta values above the significance thresholds in each of the two-group comparisons are shown. P-value for regression analysis fold changes <0.001; PADE Delta significance threshold for FA vs 200 ppb two-group comparison = 0.7; PADE Delta significance threshold for 100 ppb vs 200 ppb two-group comparison = 0.5; PADE Delta significance threshold for FA vs 100 ppb two-group comparison = 0.2. Genes highlighted in bold represent the DEGs whose expressions were also up-regulated in the separate preliminary study of 3 subjects (see Methods and S2 Table).

Post-hoc pair-wise comparison of gene expression data using PADE analysis showed 100 and 42 DEGs with a FDR ranging from <0.001 to 0.05 and from 0.04 to 0.11 across 0 to 200 ppb and 100 to 200 ppb exposures, respectively (S4 and S5 Tables). The PADE pairwise analysis of the 0 to 100 ppb exposure comparison produced a large number of DEGs at a high false discovery rate (FDR) of 0.88 (data not shown). Altogether, the PADE pairwise analyses showed the expression of 30 genes to be consistently regulated among all three exposure conditions (0 to 100 ppb; 0 to 200 ppb; and 100 to 200 ppb), all with PADE delta values above the significance thresholds in each of the two-group comparisons (Table 3). The PADE analyses did not show any statistically significant down-regulated genes for any of the comparisons.

Overall, a total of 45 genes were identified as differentially expressed using linear regression approach, a pair-wise comparison approach, or both (Table 3). Table 3 also shows the DEGs that were also up-regulated in the independent preliminary BAL cell gene expression study of separate group of subjects with 0 and 200 ppb ozone exposures as described above in the Methods section (S2 Table).

### Associated Ontologies of Upregulated Genes

**Fig 3:**
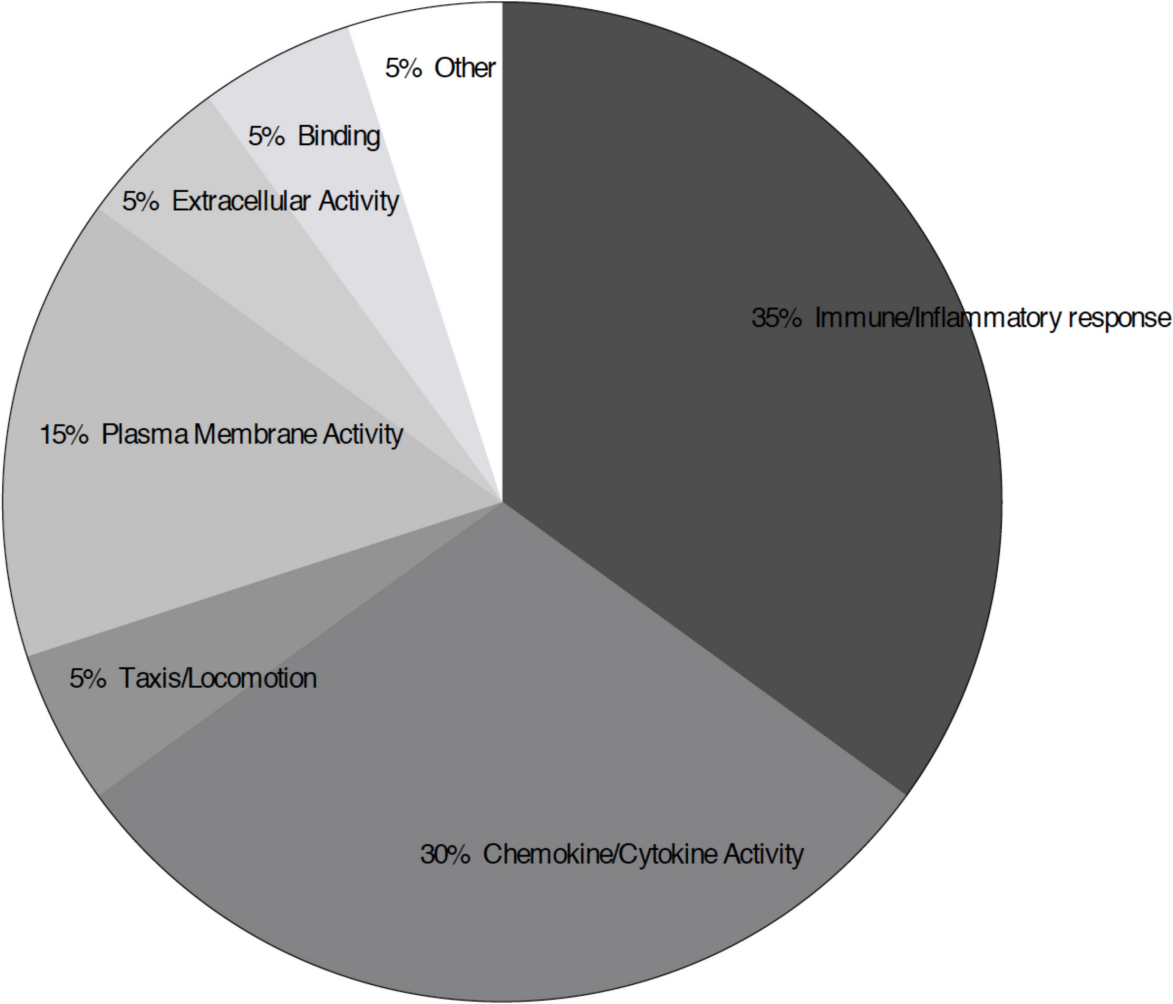
Molecular Function of Processes Associated with the Differentially Expressed Genes (DEGs) After Ozone Exposure. Analysis using DAVID showed 20 enriched gene ontologies to be associated with the DEGs based on linear regression and two-group comparison analyses with FDR threshold <25%. The gene ontologies are categorized into groups by similar molecular function.

We used DAVID to examine enriched gene ontologies associated with ozone-induced oxidative stress in BAL cells. Overall, 38 of the 45 DEGs after ozone exposure [by both regression and pair-wise (consistent across all 3 pair comparisons) analyses] were found to be involved in 20 biologic processes, including chemokine and cytokine secretion, activity, and receptor binding; adhesion, locomotion, and migration cascades; inflammatory response; extracellular space; and cell growth and tumorigenesis regulation (FDR threshold <25%) (Table 4; Fig 3). Separate analysis of the genes that were significantly up-regulated by regression or by pair-wise comparison only produced similar results as that observed in both groups.

**Table 4:** Enriched Gene Ontologies From Differentially Expressed Genes Across Ozone Exposures.

Term	Gene Descriptors	FDR (%)	p-value
GO:0006952~defense response	S100A12, CCL22, LILRA1, CORO1A, CCL2, CCR2, CLEC5A, PLA2G7, CX3CR1, SPP1, IL8RA	0.01	8.6 x 10^–6^
GO:0006955~immune response	GPR183, CCL22, CD1E, LILRA1, CD1C, CORO1A, CCL2, IL1R2, CCR2, CLEC5A, IFITM2	0.03	2.4E x 10^–5^
GO:0006935~chemotaxis	CCL22, CORO1A, CCL2, CCR2, CX3CR1, IL8RA	0.1	1.0 x 10^–4^
GO:0007626~locomotory behavior	CCL22, ANKH, CORO1A, CCL22, CCR2, CX3CR1, IL8RA	0.2	1.4 x 10^–4^
GO:0006954~inflammatory response	S100A12, CCL22, CCL2, CCR2, PLA2G7, SPP1, IL8RA	0.5	3.5 x 10^–4^
hsa04062:Chemokine signaling pathway	CCL22, CX3CR1, CCR2, PRKCB, CCL22, IL8RA	0.5	6.3 x 10^–4^
GO:0009611~response to wounding	S100A12, CCL22, CCL2, CCR2, PLA2G7, CX3CR1, SPP1, IL8RA	1.2	8.2 x 10^–4^
GO:0004950~chemokine receptor activity	CCR2, CX3CR1, IL8RA	2.0	1.7 x 10^–3^
GO:0016021~integral to membrane	ANKH, IL1R2, CLEC5A, MGAM, IFITM2, CD1E, LAMP3, SelectinL, PLXNC1, ACPP, SEMA4A, CHST15, CX3CR1, GPR183, MERTK, C4orf18, SELL, HSD11B1,	2.4	2.2 x 10^–3^
PIRSF038545:chemokine receptor	CCR2, CX3CR1, IL8RA	2.5	2.8 x 10^–3^
hsa04060:Cytokine-cytokine receptor interaction	CCL2, CCL22, IL1-R2, CCR2, CX3CR1, IL8RA	2.6	2.9 x 10^–3^
GO:0019955~cytokine binding	IL1-R2, CCR2, CX3CR1, IL8RA	2.8	2.4 x 10^–3^
GO:0005886~plasma membrane	S100A12, GPR183, MERTK, ANKH, CORO1A, SELL, PLA2G7, CCR2. CLEC5A, MGAM, PRKCB, IL8RA, KCNJ15, CD1E, LILRA1, CD1C, SelectinL, SIGLEC10	5.6	5.1 x 10^–3^
IPR007110:Immunoglobulin-like	CD1E, MERTK, LILRA1, CD1C, IL1R2, SIGLEC10	9.1	8.2 x 10^–3^
GO:0005887~integral to plasma membrane	GPR183, CD1E, MERTK, ANKH, CD1C, SELL, CCR2, CLEC5A, SelectinL, CX3CR1, KCNJ15	10.4	9.8 x 10^–3^
IPR013151:Immunoglobulin	MERTK, LILRA1, IL1R2, SIGLEC10	15.4	1.4 x 10^–2^
GO:0006968~cellular defense response	CCR2, CLEC5A, CX3CR1	17.9	1.4 x 10^–2^
GO:0005615~extracellular space	CCL22, CCL2, PLA2G7, SULF2, MMP9, SPP1, MMP8	19.1	1.9 x 10^–2^
GO:0015674~di-, tri-valent inorganic cation transport	CORO1A, STEAP4, PRKCB, SLC25A37	19.2	1.5 x 10^–2^
GO:0007155~cell adhesion	CORO1A, CCL2, SELL, SIGLAC10, PLXNC1, CX3CR1, SPP1	20.3	1.6 x 10^–2^

Analysis using DAVID showed 20 enriched gene ontologies to be associated with the DEGs based on linear regression and pair-wise comparison (consistent across all 3 pairs) analyses with FDR threshold <25%.

In addition, we used DAVID to examine gene ontology of the DEGs of 0 and 200 ppb pairwise comparison, which represented the DEGs from the largest ozone exposure signal. This analysis identified 16 biologic processes with FDR threshold <25% including cytokine binding and interaction; chemotaxis and locomotory behavior; inflammatory response; extracellular space and region; and response to wounding, with involvement of 18 out of the 100 DEGs (Table 5).

**Table 5:** Enriched Gene Ontologies From Differentially Expressed Genes Across 0 and 200 ppb Ozone Exposure.

Term	Gene Descriptors	FDR (%)	p-value
GO:0005615~extracellular space	IL8, CCL2/mcp-1, HTRA1, PLA2G7, SULF2, MMP9, SPP1, MMP8	0.3	2.5 x 10^–4^
GO:0044421~extracellular region part	IL8, CCL2/mcp-1, HTRA1, PLA2G7, PI3, SULF2, MMP9, SPP1, MMP8	0.3	3.4 x 10^–4^
hsa04060:Cytokine-cytokine receptor interaction	IL8, CCL2/mcp-1, IL1R2/CD121b, HGF, CX3CR1, IL8RA/CXCR1/CD181	0.4	4.8 x 10^–4^
GO:0006954~inflammatory response	S100A12, IL8, CCL2/mcp-1, PLA2G7, SPP1, IL8RA/CXCR1/CD181	0.4	2. x 10^–4^
GO:0009611~response to wounding	S100A12, IL8, CCL2/mcp-1, PLA2G7, CX3CR1, SPP1, IL8RA/CXCR1/CD181	0.5	3.3 x 10^–4^
GO:0006952~defense response	S100A12, IL8, CCL2/mcp-1, PLA2G7, CX3CR1, SPP1, IL8RA/CXCR1/CD181	1.0	7.3 x 10^–4^
GO:0005576~extracellular region	IL8, SPINK1, CCL2/mcp-1, HTRA1, HGF, PLA2G7, PI3, SULF2, MMP9, SPP1, MMP8	3.1	3.0 x 10^–3^
GO:0006935~chemotaxis	IL8, CCL2/mcp-1, CX3CR1, IL8RA/CXCR1/CD181	4.1	3.1 x 10^–3^
GO:0042330~taxis	IL8, CCL2/mcp-1, CX3CR1, IL8RA/CXCR1/CD181	4.1	3.1 x 10^–3^
hsa04062:Chemokine signaling pathway	IL8, CCL2/mcp-1, CX3CR1, IL8RA/CXCR1/CD181	10.0	1.3 x 10^–2^
GO:0006955~immune response	IL8, GPR183, CD1E, CD1C, CCL2/mcp-1, IL1R2/CD121b	10.1	7.7 x 10^–3^
GO:0019955~cytokine binding	IL1R2/CD121b, CS3CR1, IL8RA/CXCR1/CD181	15.3	1.4 x 10^–2^
hsa04640:Hematopoietic cell lineage	CD1E, CD1C, IL1R2/CD121b	16.5	2.3 x 10^–2^
GO:0007626~locomotory behavior	IL8, CCL2/mcp-1, CX3CR1, IL8RA/CXCR1/CD181	17.1	1.3 x 10^–2^
GO:0004175~endopeptidase activity	HTRA1, HGF, MMP9, MMP8	24.6	2.4 x 10^–2^

Analysis using DAVID showed 15 enriched gene ontologies to be associated with the DEGs based on pair-wise comparison between 0 and 200 ppb ozone exposure with FDR threshold <25%.

Furthermore, Ingenuity Pathway Analysis was also used to examine Gene Set Analysis (GSA) of differential regulation of Biocarta, Gene Ontology, and Kyoto Encyclopedia of Genes and Genomes (KEGG) processes or genes after ozone exposure (Table 6). Overall, 17 pathways were found to have a GSA FDR threshold <5%. In addition to the processes identified using DAVID, GSA detected several other processes, including metallopeptidase activity and epidermal growth factor (EGF) processes involved in tissue repair and remodeling. S6 Table shows all pathways identified using GSA with FDR threshold <25%.

**Table 6:** Gene Set Analysis (GSA) Using Ingenuity Pathway Analysis (IPA).

Set Name	GSA Score	GSA p-value	GSA FDR (%)
KEGG_HEMATOPOIETIC_CELL_LINEAGE	0.634	<0.001	<0.1
BIOCARTA_CALCINEURIN_PATHWAY	0.688	<0.001	<0.1
BIOCARTA_CCR5_PATHWAY	0.857	<0.001	<0.1
KEGG_CYTOKINE_CYTOKINE_RECEPTOR_INTERACTION	0.385	<0.001	<0.1
G_PROTEIN_COUPLED_RECEPTOR_BINDING	0.667	<0.001	<0.1
METALLOPEPTIDASE_ACTIVITY	0.457	<0.001	<0.1
CHEMOKINE_ACTIVITY	0.684	<0.001	<0.1
CHEMOKINE_RECEPTOR_BINDING	0.787	<0.001	<0.1
METALLOENDOPEPTIDASE_ACTIVITY	0.647	<0.001	<0.1
BEHAVIOR	0.499	<0.001	<0.1
LOCOMOTORY_BEHAVIOR	0.690	<0.001	<0.1
INFLAMMATORY_RESPONSE	0.422	<0.001	<0.1
RESPONSE_TO_EXTERNAL_STIMULUS	0.352	<0.001	<0.1
RESPONSE_TO_WOUNDING	0.415	<0.001	<0.1
KEGG_ALDOSTERONE_REGULATED_SODIUM_REABSORPTION	0.483	0.002	4.5
BIOCARTA_CARDIACEGF_PATHWAY	0.651	0.002	4.5
KEGG_CHEMOKINE_SIGNALING_PATHWAY	0.287	0.002	4.5

GSA identified 17 processes associated with the DEGs from linear regression analysis with FDR threshold <5%.

### Cluster Analysis of Gene Expression Based on Clinical Covariates

We used hierarchic clustering to group BAL cell samples based on the pattern of expression of the DEGs without regard to whether their expression was different based on the clinical covariates examined. Although above the pre-specified FDR threshold, at p-value of 0.01 and 1.5-fold change, both asthma status and lung function response to ozone showed clustering effects of gene expression at the 200 ppb level of exposure. Asthmatic subjects and non-asthmatic subjects grouped together in two separate clusters with only 3 non-asthmatic subjects clustering with the asthmatic subjects (Fig 2 part B). Similarly, lung function responders and non-responders grouped together in two separate clusters with only 2 non-responders clustering with the responders (Fig 2 part C). No significant clustering effect was found for sex, atopy, or neutrophilic or eosinophilic response status. There was no significant clustering effect for any of the covariates at the 100 ppb level of exposure.

### Effects of Stratification by Covariates on Gene Expression

**Fig 4:**
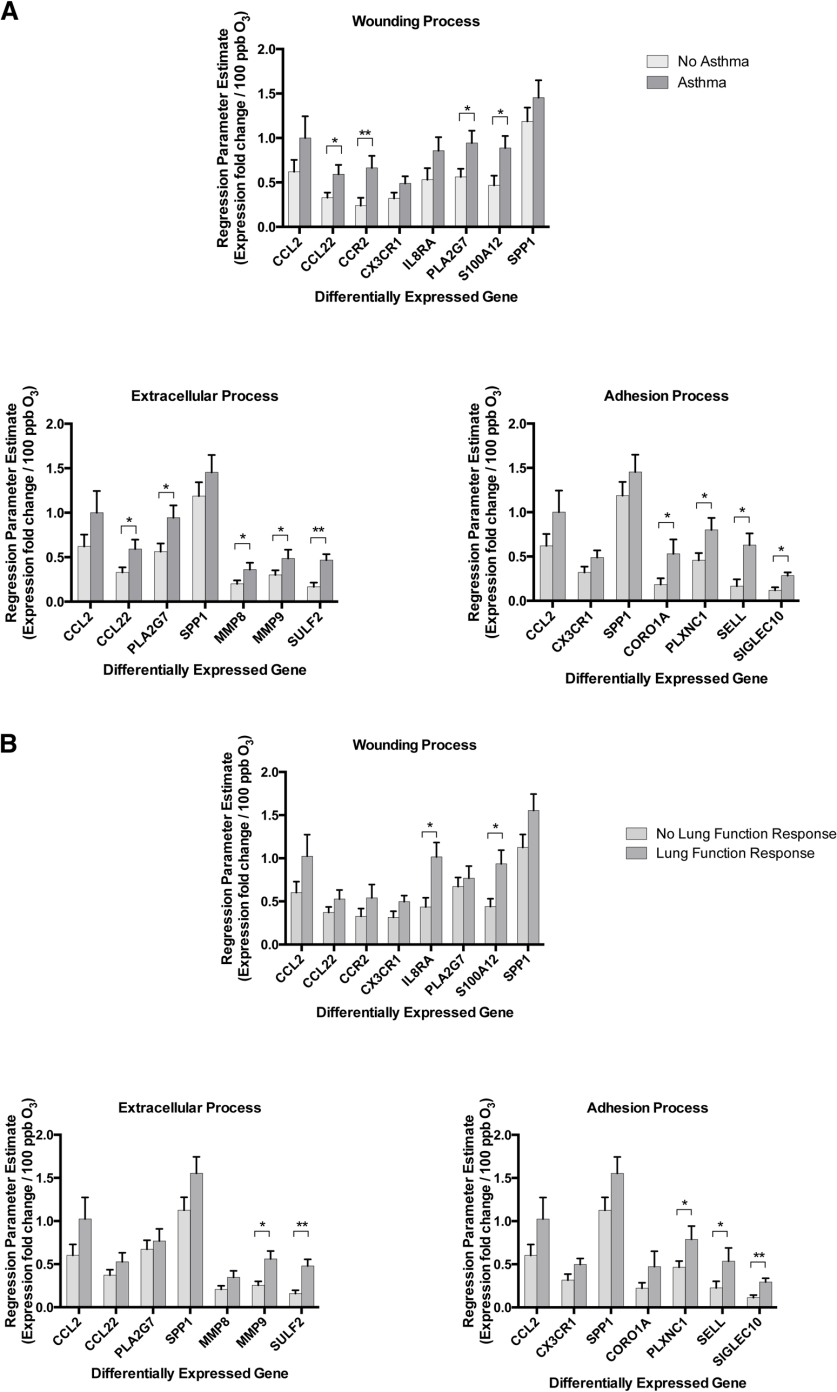
Stratification of BAL Cells Gene Expression After Ozone Exposure by Covariates. Rate of increase in expression (parameter estimate ±SEM in fold change per 100 ppb increase in level of ozone exposure) of DEGs involved in some of the identified processes from DAVID with stratification for **(A)** asthma status or **(B)** lung function response to ozone. O_3_: ozone.

Stratification by asthma showed a significantly higher rate of up-regulation of gene expression of most, but not all, DEGs with ozone exposure (Fig 4 part A). Stratification by lung function response also showed a higher rate of up-regulation of many DEGs (Fig 4 part B). Despite the similarity in up-regulation of the DEGs with the above stratifications, there was no significant association between asthma status and lung function response to ozone among subjects (chi-squared p-value = 0.67). Stratification by neutrophilic response showed a significant change in the expression of only a single DEG (S100A12). Stratification by sex, atopy, or eosinophilic response did not reveal any differences in the rate of expression of the DEGs.

### Interaction Network Analysis of Upregulated Genes

To infer the possible relationships and interactions between the up-regulated genes after exposure to ozone, we examined the DEGs that were identified from the 0 to 200 ppb ozone pair-wise comparison using Ingenuity iReport. Overall, 49 DEGs (q-value <0.05 and fold change >1.5) were identified to be up-regulated, and 1 gene was identified to be down regulated (RNU-4) (q-value of 0.0017 and fold change of -1.56). Using these DEGs, several relevant pathways, processes, diseases, and interactions were detected (Supplemental S7, S8, and S9 Tables). Granulocyte and agranulocyte adhesion/diapedesis were found to be the most relevant pathways (p-value of 1.93x10^-8^ and 3.12x10^-8^, respectively), each involving 8 DEGs. A number of other processes similar to those identified using DAVID and GSA were also detected. CCL2, CCR2, and SPP1, the top three genes from this analysis, were each associated with over 150 processes. Eight DEGs did not have any associated processes. S2 Fig shows the network of 13 DEGs and their reported interactions, which are organized by downstream or upstream activity relative to the other DEGs in the dataset. All DEGs listed in the proposed interaction are up-regulated after exposure to ozone. The remaining 37 DEGs had low connectivity to each other and had zero interacting neighbors within the dataset.

### Osteopontin RNA Expression in BAL Cells and Protein in BAL are Up-regulated Following Exposure to Ozone

**Fig 5:**
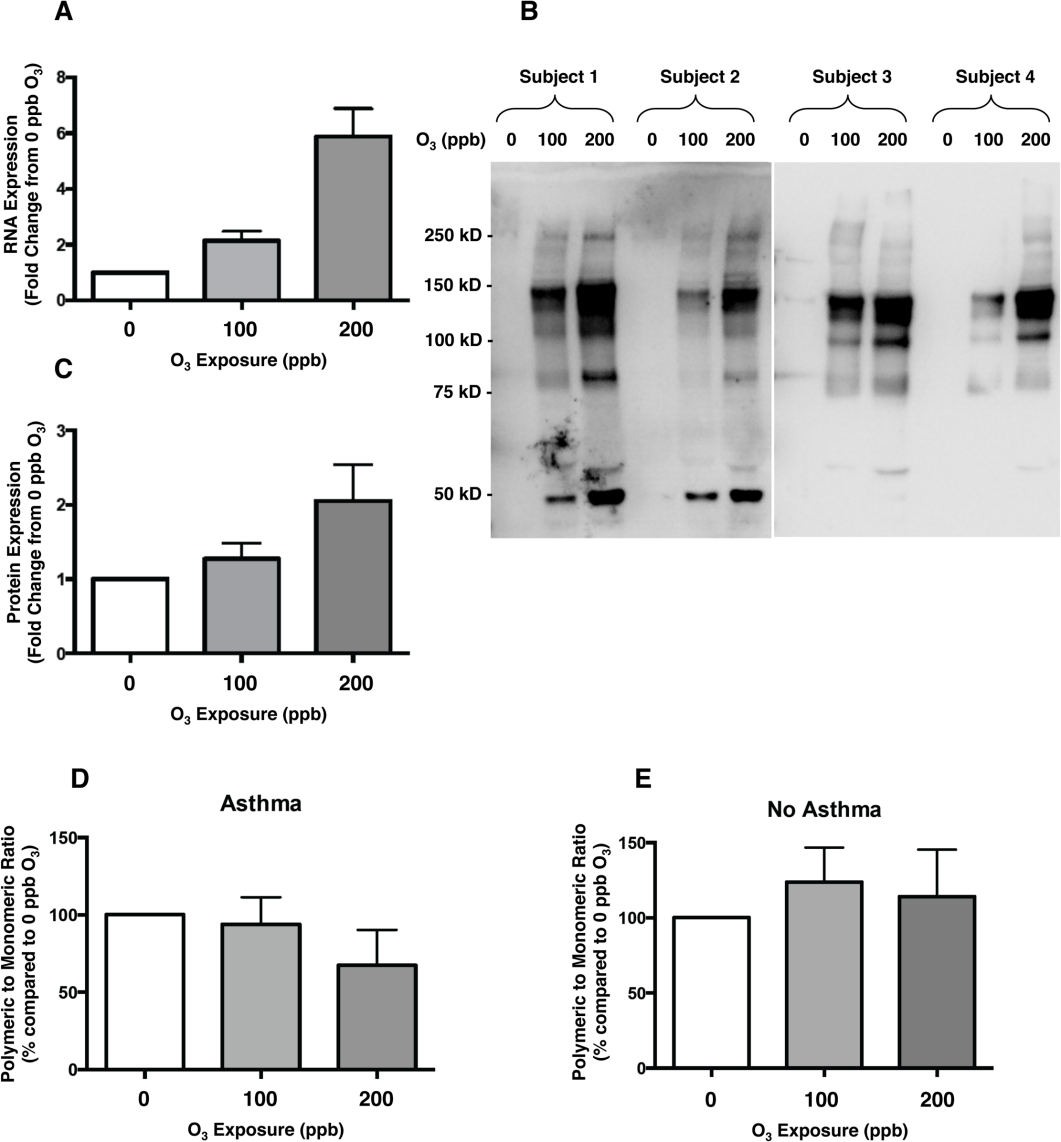
Expression of Osteopontin RNA and Protein in BAL After Ozone Exposure. **A.** Osteopontin gene (SPP1) RNA expression increased with increasing ozone exposure (q-value of 9.1x10^-7^ in linear regression analysis). **B.** Immunoblot of BAL samples from four representative subjects after exposure to 0, 100, and 200 ppb ozone probed with anti-OPN antibody. **C.** Osteopontin total protein (monomeric and polymeric forms) signal (from immunoblot densitometry analysis) increased with increasing ozone exposure (linear regression p-value = 0.03). **D & E.** The ratio of polymeric to monomeric forms of osteopontin decreased with increasing ozone exposure in those subjects with asthma (linear regression p-value = 0.01). Bars represent Mean ± SEM.

The most highly DEG after ozone exposure by either linear regression or pair-wise analysis was secreted phosphoprotein 1 (SPP1), the gene for osteopontin (OPN), corroborating the same finding in our independent preliminary gene expression study of a separate group of subjects (Supplemental S2 Table). SPP1 expression displayed a 2.1- and 6.0-fold up-regulation from baseline 0 ppb exposure at the 100 ppb and 200 ppb levels of ozone exposure, respectively (q-value of 9.1x10^-7^ in linear regression analysis) (Table 3; Fig 5 part A).

Immunoblotting of BAL fluid after ozone exposure confirmed a dose-dependent increase in OPN protein level, including both monomeric (~55–60 kDa) and polymeric (>60 kDa) forms (Fig 5 parts B and C). The total OPN protein level in BAL fluid was associated with the level of exposure to ozone [parameter estimates±standard error of mean (PE±SEM) of 1.28±0.43 fold increase per 100 ppb ozone (p = 0.003)]. Both monomeric and polymeric forms of OPN protein contributed to this increase in total OPN level [PE±SEM of 0.85±0.26 (p = 0.001) and 0.43±0.21 (p = 0.04) fold increase per 100 ppb ozone, respectively]. While there was no significant change in the ratio of polymeric to monomeric OPN in all subjects across exposures, in those with asthma, there was a significant decrease in the ratio of polymeric to monomeric OPN with increasing ozone exposure [PE±SEM of -14.5±8.9% (p = 0.01) and 10.7±12.0% (p = 0.81) change in ratio of polymeric to monomeric OPN per 100 ppb ozone for those with and without asthma, respectively] (Fig 5 parts D and E).

### Polymeric OPN Enhanced Wound Closure in a Human Airway Epithelial Cell Line

**Fig 6:**
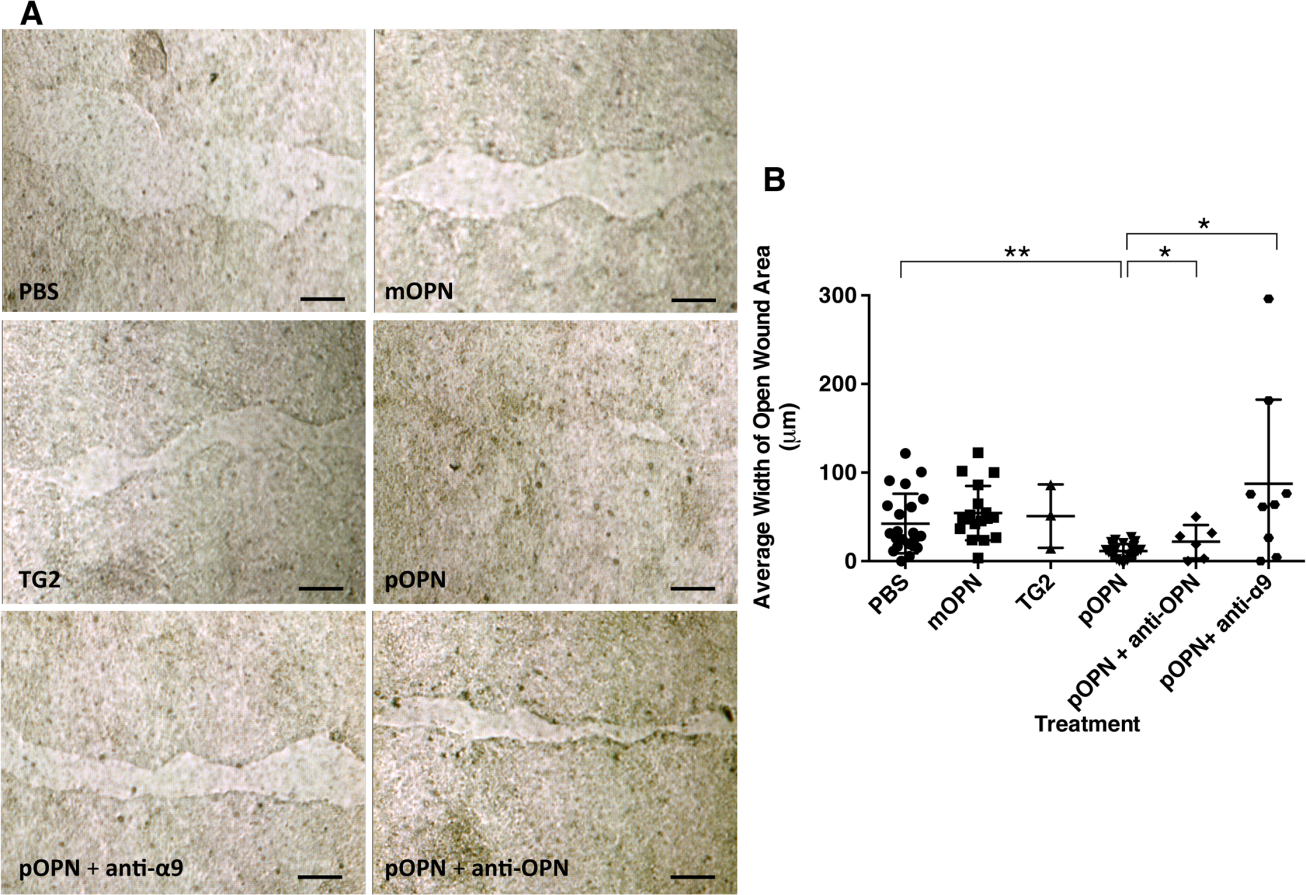
Effect of Osteopontin on Epithelial Wound Closure. 16HBE14o- cells were grown to confluence on trans-well membrane plates and used in a scratch assay as a model of wound closure. **A.** Open wound area was measured 14 hours after scratch and treatment with saline (PBS), monomeric osteopontin (mOPN), transglutaminase 2 (TG2), polymeric osteopontin (pOPN), with and without anti-osteopontin antibody (anti-OPN) or anti-α9 integrin antibody (anti-α9). The scale bar is 50 μm. **B.** Average width of open wound area was calculated by adjusting wound area for length of scratch. The graph shows mean (middle horizontal bar) and standard deviation (whiskers).

OPN has been reported to play a role in tissue repair and remodeling in animal models including a described “altered” response to lung fibrosis in bleomycin-induced lung injury [43–45]. To investigate the role of OPN and its post-translational modification in human airway epithelial repair, we examined its effect in wound repair using an *in vitro* model of injury (scratch assay) in 16HBE14o- normal human bronchial epithelial cell line. Treatment with monomeric OPN (mOPN) did not significantly affect wound closure at the 14-hour time point compared to treatment with buffer (PBS) alone [decrease in wound area closure of (mean±SEM) 28.0±24.2%; p = 0.26] (Fig 6). However, treatment with polymeric OPN (pOPN) led to increased wound closure at the 14-hour time point compared to treatment with buffer alone [increase in wound area closure of 73.3±17.0%; p = 0.0001]. Addition of anti-OPN blocking antibody (R&D AF1433) to pOPN treatment nullified its effect on wound closure [decrease in wound area closure of 25.2±11.8%; p = 0.04 compared to pOPN treatment alone], and was no different compared to treatment with buffer alone. Treatment with TG2 or anti-OPN blocking antibody alone did not significantly affect wound closure.

### Effect of Polymeric OPN on Wound Closure was α9β1 Integrin-dependent

Flow cytometry showed the presence of multiple cell surface integrins on 16HBE14o- cells, including α9 and β1 integrins (S2 Fig part A). The presence of α9 integrin, which exclusively dimerizes with β1 integrin to form α9β1 heterodimer receptor, was further confirmed by immunoblot assay of 16HBE14o- cell lysate using anti-α9 antibody (S2 Fig part B). To determine whether the effect of pOPN on wound closure was mediated by α9β1 integrin, 16HBE14o- cells were treated with anti-α9 blocking antibody (Y9A2) and pOPN. Addition of anti-α9 blocking antibody to pOPN treatment nullified its effect on wound closure [decrease in wound area closure of 279.0±78.2%; p = 0.0012 compared to pOPN treatment alone] (Fig 6).

## Discussion

In this study, we found that inhalation of medium and high ambient levels of ozone causes a dose-dependent up-regulation of a relatively small collection of genes in human BAL cells. The ozone-induced up-regulated genes map to several biologic processes such as cytokine and chemokine signaling, cell adhesion, and extracellular space modification, processes that are involved in cell trafficking, inflammation, and tissue repair and remodeling. The pattern of gene expression of BAL cells after ozone exposure was different between those with and without asthma, and between those with and without lung function response (so-called “ozone responders”). In particular, having even mild asthma and a lung function response to ozone was associated with a 1.7- to 3.8-fold and a 1.7- to 2.6-fold higher rate of expression in many of the DEGs, respectively. However, there was no distinction in DEGs between those with a higher neutrophilic or eosinophilic inflammatory response to ozone and those with a lower response.

Despite the similar BAL neutrophilic and eosinophilic response to ozone exposure between subjects with and without asthma, those with asthma had distinct higher up-regulation of many of DEGs involved in wounding, adhesion, and extracellular processes, with an increased pro-inflammatory signal (PLA2G7, S100A12, CORO1A, PLXNC1, SELL, and SIGLEC10), a predilection for M1 macrophage phenotype (CCR2 and CCL22), and augmented matrix degradation and remodeling (MMP8, MMP9, and SULF2). These findings are consistent with an earlier report on gene expression of airway cells obtained with sputum sampling after exposure to higher doses of ozone (400 ppb for 2 hours with sampling at 4 hours after the exposure) [46]. Although only a small number of BAL cell DEGs that we identified were also reported to be differentially expressed in the sputum airway cell samples, many of the processes identified, particularly the increased pro-inflammatory signals, were similar. Interestingly, both our study of BAL cells and the study of sputum airway cells identified increased expression of processes associated with innate immunity in subjects with asthma versus those without. The differences in DEGs observed between our study and the sputum study are not necessarily unexpected because although both BAL and sputum are valid methods of sampling airways, the inflammatory endpoints they measure are not closely correlated [24]. Notably, BAL cells may better represent a sample of the distal lung. In addition, the differences in exposure levels and the time frame of sampling may have contributed to the differences observed.

Next, we identified a secreted protein, OPN, whose gene (SPP1) was the most highly expressed gene in BAL cells after ozone exposure, and whose protein level in BAL showed a corresponding dose-dependent increase with ozone exposure. OPN is a heavily phosphorylated glycoprotein with several ascribed functions in tissue repair and remodeling, fibrosis, mineralization, and immunomodulation, as well as inflammation [47]. It is a chemokine for macrophages and neutrophils and plays a role in adhesion and migration of various cell types. OPN has alternate splicing variants, and undergoes considerable tissue-specific post-translational modifications, including polymerization by tissue transglutaminase 2 (TG2) [48, 49]. The different variants of OPN are thought to be responsible for its variety of function [50, 51]. OPN is a ligand for at least nine members of the integrin family including αvβ3, αvβ5, α4β1, α4β7, and α9β1, through interaction with its RGD peptide sequence [52], and polymeric OPN has been shown to have increased affinity for α9β1 integrin [50, 51].

Given the reported differential function of OPN polymeric and monomeric forms, we examined the role of both forms in ozone-induced oxidative injury of airway epithelium in an *in vitro* injury model using a human bronchial epithelial cell line (16HBE14o-), and found that treatment with polymeric OPN, but not monomeric OPN, enhanced the epithelial wound closure in this model. We also showed the effect of OPN on our *in vitro* model of epithelial wound injury to be α9β1 integrin-dependent by demonstrating that its effect on wound closure was eliminated if the 16HBE14o- cells were pre-treated with blocking antibody against the α9 subunit of α9β1 integrin. We had previously shown the secreted OPN to be highly polymerized in human airways, and less polymeric and more monomeric and fragmented in those with asthma [28]. Here, we showed that while ozone caused a dose-dependent increase in both polymeric and monomeric forms of OPN in BAL in all subjects, in those with asthma, the ratio of polymeric to monomeric OPN declined with increasing levels of ozone exposures. Given our observation that polymeric OPN has a differential role in epithelial wound repair, the progressive decline in the ratio of polymeric to monomeric (or fragmented) OPN in asthmatic subjects may be taken as a possible explanation for the increased morbidity seen in asthmatic subjects with exposure to ambient levels of ozone. While OPN has been shown to augment migration of osteocytes and wound healing in bone tissue [43], to our knowledge, this is the first report showing that osteopontin mediates wound healing in human bronchial epithelial cells.

Several studies have examined lung expression profiles in mouse and rat models after acute exposure to ozone [53–55]; however, the reported RNA expression responses appear to be largely different compared to those that we have reported here in humans. These studies showed that acute exposure to ozone caused a distinct induction of inflammatory pathways in rat and mouse lungs, as was found in our study. Nevertheless, almost none of the genes found to be significantly up-regulated showed a parallel effect in our human study. It is important to note that while the above studies examined RNA profiles in whole lung tissue, we examined RNA profiles in BAL cells, which may contribute to the observed discrepancies. Other possible explanations are the difficulties in matching exposure doses and sampling time frames between rodent and human studies. Vasu, et al. have reported SPP1 to be up-regulated after ozone exposure (6 hours per day for 3 days to 500 ppb ozone) in lung tissue of a strain of mice with an antioxidant deficiency (Alpha-tocopherol transfer protein (ATTP) null mice) [56]. More recently, Barreno, et al. specifically explored the inflammatory role of OPN in lungs in a mouse model. They found an increase in OPN in whole lung lavage (WLL) following ozone exposure, and showed differences in WLL neutrophilia and lung resistance response to methacholine [equivalent of BAL and AHR in humans] between OPN knock out mice and their wild type background (C57BL/6J). Most interestingly, Barreno’s report showed differences between knock out and wild type mice in WLL inflammatory cells and lung resistance even at baseline without ozone exposure, an intriguing finding which we have also observed in experimentation with these knock out mice (unpublished data). The implication of these baseline differences is unclear, but it may underscore potential pro-inflammatory and pro-fibrotic roles for OPN. It is important to note that although the Barreno, et al. findings suggest that OPN plays a role in mediating inflammation, the functions of different variants of OPN, including intracellular (iOPN), alternate splicing (OPN-a, OPN-b, OPN-c), and post-translational modification (polymerization and phosphorylation) forms, were not examined. Other investigators have reported disorganized collagen matrix underlying pulmonary arteries in OPN knock out mice in the absence of any inciting stimulus, which suggests a role for OPN in collagen metabolism and tissue modeling in mice [57]. Our *in vitro* experiments corroborate the above report and suggest a role for the polymeric form of OPN in bronchial epithelium wound repair.

There are a few potential limitations to our study. First, we did not use a specific cell population of BAL cells and instead pooled all cell populations obtained from the lavage to examine gene expression of BAL cells. While BAL samples were composed of different cell types, the goal of this study was not to see whether gene expression in any particular cell population changed, but rather how expression response changed collectively. Interestingly, although ozone exposure caused a significant neutrophilia in BAL as expected (the hallmark of ozone-induced airway inflammation), stratification of gene expression by neutrophilia did not show any difference between subjects with high and low neutrophilic response. Additionally, the concentration of non-inflammatory cells, including epithelial cells, an important group of cells given the goal of this study to examine the injury and repair processes, did not significantly change across exposures. Thus, the gene expression trends that were observed in this study are unlikely to be due to changes in the composition of BAL cells with ozone exposure. Second, the gene expression data obtained in our study reflects the expression pattern of airway inflammatory cells after exposure to ozone at the 24-h time point. It is acknowledged that the gene expression of these cells in an earlier or later time point after ozone exposure may be considerably different than at the 24-h time point. Previous studies have shown that ozone-induced granulocytic inflammation peaks at 6 hours, persists to about 18 to 20 hours, and then attenuates at 24 hours [11], and thus, the 24-h time point is a reasonable time during which resolution of inflammation and tissue repair processes may be active. For example, IL-6, which is one of the acute phase response cytokines that has been consistently identified to be increased with ozone exposure, was not identified as a DEG in this study (although its BAL protein level was increased linearly with increasing ozone exposure [data not shown]). Two possible explanations for this observation are: (1) IL-6 gene expression increases early after ozone exposure resulting in an increase in IL-6 protein production and secretion but at the 24-h time point its gene expression may be attenuated while its protein level in BAL remains elevated; (2) IL-6 is known to be expressed, synthesized, and secreted into airway by cells (airway epithelial and smooth muscle cells) [58, 59] other than BAL cells whose gene expression is examined here. Third, we used an *in vitro* scratch assay to assess a potential function for the most highly expressed gene (SPP1) after ozone-induced oxidative injury. While this scratch assay may not be a model of ozone-induced oxidative injury in epithelial cells, it has been extensively used as a model of epithelial injury. Further experiments with models of injury that use *in vitro* ozone exposure would provide more robust evidence on the role of osteopontin in repair of epithelium after ozone inhalation. However, the current experiment does provide evidence of its role in a well-established model of injury in epithelium. Lastly, our wound assay did not entail a real-time examination of rate of wound closure using multiple time-point measurements. However, the 14-hour end-point measurement was optimized to detect significant changes in wound closure in our model.

## Conclusion

In conclusion, our findings show that (1) inhalation of ozone leads to a dose-dependent up-regulation of several biologic pathways involved in inflammation and repair including chemokine and cytokine secretion, activity, and receptor binding; metalloproteinase and endopeptidase activity; adhesion, locomotion, and migration; and cell growth and tumorigenesis regulation; (2) asthmatic subjects had higher expression of many DEGs suggestive of increased proinflammatory and matrix degradation and remodeling signals; (3) the most highly up-regulated gene was osteopontin, the protein level of which in BAL fluid increased in a dose-dependent manner after ozone exposure; (3) subjects with asthma have a disproportionate increase in non-polymerized osteopontin with exposure to increasing levels of ozone compared to those without asthma; and (3) polymeric, and not monomeric, OPN enhances wound closure in an *in vitro* model of epithelial injury in an α9β1 integrin-dependent manner.

## Supporting Information

S1 Fig Biologic Connectivity of Some of the Differentially Expressed Genes (DEGs) After Ozone Exposure. Thirteen differentially expressed genes (DEGs, p-value <0.05 and fold change >1.5) showed connectivity within the iReport results dataset. The diagram shows the relative connectivity of the DEGs based on their known upstream or downstream activity.
(TIF)

S2 Fig Flow Cytometry of Cell Surface Integrins and Immunoblot of 16HBE14o- Cell Lysates. **A**. Flow cytometry showed α9, β1, β5 and β6 integrins to be present on the surface of 16HBE14o- cells. **B**. Immunoblot assay of 16HBE14o- cell lysates using anti-α9 integrin antibody showed presence of this integrin in the cell lysates.
(TIF)

S1 Methods Supplemental Methods. (DOCX)

S1 Table Exposure Randomization. Data presented as the number of subjects in each exposure-order assignment. Pearson chi-squared p-value = 0.09.
(DOCX)

S2 Table Preliminary Differential Gene Expression from Pilot Study. Differentially expressed genes from a previous preliminary study of 3 subjects exposed to 0 and 200 ppb ozone [35]. B-score: log posterior odds ratio statistic, where odds ratio is the ratio between the probability that a given gene is differentially expressed over the probability that it is not differentially expressed (a negative B value means that the gene has a bigger chance of not being differentially expressed than being differentially expressed); FDR: false discover rate; p-values were adjusted using Holm method. Genes highlighted in bold were selected *a priori* for their role in migration, tissue repair and remodeling, immune response, inflammation, and extracellular region (see Methods).
(DOCX)

S3 Table Exposure Conditions. Data presented as mean±SD. a, b, c: indicate pair-wise comparisons with significant differences (p<0.05). V_E_: average minute ventilation during exercise; O_3_: ozone.
(DOCX)

S4 Table Differentially Expressed Genes with PADE pair-wise Analysis from 0 to 200 ppb Ozone Exposure. The significant differentially expressed genes from PADE pair-wise analysis are listed with PADE delta values and fold changes. PADE Delta significance threshold for 0 versus 200 ppb pair-wise comparison = 0.7. Genes highlighted in bold represent the DEGs whose expressions were also up-regulated in the separate preliminary study of 3 subjects (see Methods and Supplemental S2 Table).
(DOCX)

S5 Table Differentially Expressed Genes with PADE pair-wise Analysis from 100 to 200 ppb Ozone Exposure. The significant differentially expressed genes from PADE pair-wise analysis are listed with PADE delta values and fold changes. PADE Delta significance threshold for 100 versus 200 ppb pair-wise comparison = 0.5.
(DOCX)

S6 Table Gene Set Analysis (GSA) Using Ingenuity Pathway Analysis (IPA). GSA identified several processes associated with the DEGs from linear regression analysis with FDR threshold <25%.
(DOCX)

S7 Table Pathways Associated with the DEGs by iReport. iReport generated 19 pathways that were associated with the 49 DEGs from the two-group comparison of 0 to 200 ppb ozone exposure. Pathways with a p-value < 0.01 are shown.
(DOCX)

S8 Table Processes Associated with the DEGs by iReport. iReport generated 59 processes that were associated with the 49 DEGs from the two-group comparison of 0 to 200 ppb ozone exposure. Pathways with a p-value <1x10^-8^ are shown.
(DOCX)

S9 Table Diseases Associated with the DEGs by iReport. iReport generated 25 diseases that were associated with the 49 DEGs from the two-group comparison of 0 to 200 ppb ozone exposure. Diseases with a p-value <1x10^-6^ are shown.
(DOCX)
