# Supplementary figures and images for: Inflammatory and Repair Pathways Induced in Human Bronchoalveolar Lavage Cells with Ozone Inhalation
Source: PLoS One. 2015 Jun 2;10(6):e0127283. doi: 10.1371/journal.pone.0127283 (PMC4452717; doi:10.1371/journal.pone.0127283)

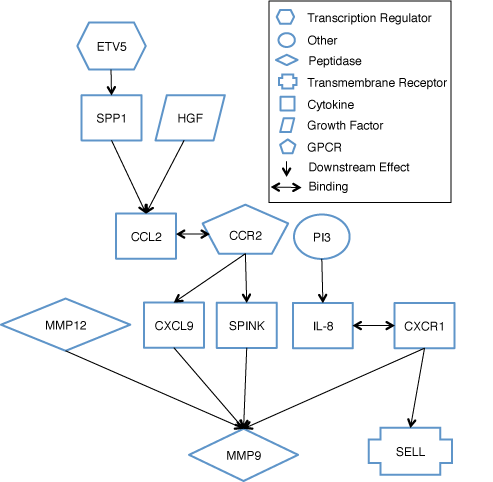

Supplement: S1 Fig — Thirteen differentially expressed genes (DEGs, p-value <0.05 and fold change >1.5) showed connectivity within the iReport results dataset. The diagram shows the relative connectivity of the DEGs based on their known upstream or downstream activity. (TIF) [file pone.0127283.s001.tif]

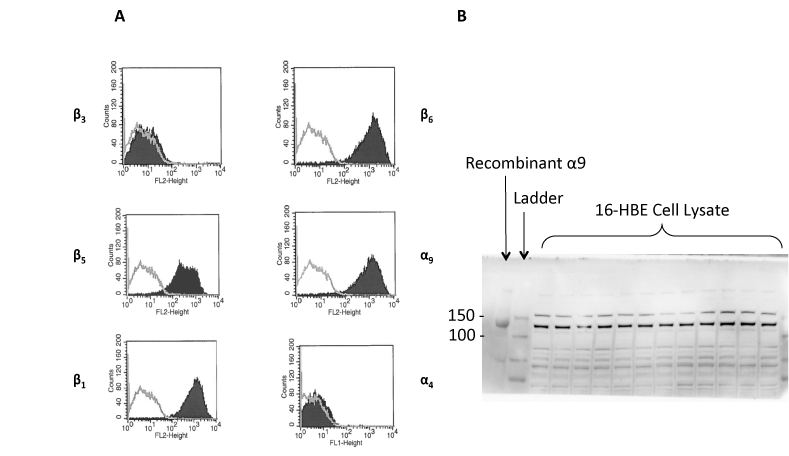

Supplement: S2 Fig — A. Flow cytometry showed α9, β1, β5 and β6 integrins to be present on the surface of 16HBE14o- cells. B. Immunoblot assay of 16HBE14o- cell lysates using anti-α9 integrin antibody showed presence of this integrin in the cell lysates. (TIF) [file pone.0127283.s002.tif]
