# Supplementary material for: Inflammatory and Repair Pathways Induced in Human Bronchoalveolar Lavage Cells with Ozone Inhalation
Source: PLoS One. 2015 Jun 2;10(6):e0127283. doi: 10.1371/journal.pone.0127283 (PMC4452717; doi:10.1371/journal.pone.0127283)
Supplement: S1 Methods — (DOCX) [file pone.0127283.s003.docx]

**S1 METHODS**

**Spirometry**

Each subject’s spirometry and peak expiratory flow were measured immediately before (0-h) and immediately after (4-h) exposure, and immediately before bronchoscopy (24-h). Spirometry was performed on a dry rolling-seal spirometer (PDS, nSpire Health, Inc., Longmont, CO) following American Thoracic Society performance criteria [[60](#_ENREF_60)]. The best values for FVC and FEV_1_ from three acceptable FVC maneuvers were used in data analysis.

**In vitro Polymerization of rOPN by Transglutaminase 2**

Polymeric OPN was synthesized from recombinant monomeric OPN (R&D Systems, Minneapolis, MN) using 20 ng/ml of tissue transglutaminase 2 (TG2) (R&D Systems) in a buffer containing CaCl_2_, Tris, and DTT as described previously [[28](#_ENREF_28)].

**Western Blot Materials**

Goat affinity-purified anti-human OPN polyclonal antibody (AF1433) raised against natural human milk sOPN, anti-goat IgG-HRP antibody (AB-108-C), and human rOPN (1433-OP) made in a murine myeloma cell line were purchased from R&D Systems. BSA, Tris-HCl, and Tween 20 were obtained from Sigma-Aldrich (St. Louis, MO).

**Denaturing Western Blot**

Immunoblotting was performed using Invitrogen XCell Sure-Lock Mini-Cell and XCell II BlotModule kit and reagents and NuPAGE 4-12% gradient Bis-Tris gels (Life Technolgoies, Carlsbad, CA) as described previously [[28](#_ENREF_28)]. Briefly, BAL fluid samples were concentrated 10-fold using Amicon Ultra-15 3 kDa filters (Millipore, Billerica, MA), and 11 μL of each sample were resolved by gel electrophoresis and transferred onto a PVDF membrane. Primary antibodies against human osteopontin (R&D Systems) were diluted 1:1000 in 1% bovine serum albumin in TBS-0.1% Tween. PVDF membranes were incubated in primary antibody solutions overnight at 4°C. HRP-conjugated secondary antibodies were diluted 1:1000 in 1% bovine serum albumin in TBS-0.1% Tween and incubated with membranes for 30 minutes at room temperature. Blots were subsequently developed using Super Signal Peroxide Buffer and Enhancer Solution (Thermo Scientific, Waltham, MA), and analyzed with the Alpha Innotech Fluorchem Sp Digital Imaging System and FluorChem Sp Software (San Leandro, CA). Optical densitometry was measured using ImageJ software as described previously [[28](#_ENREF_28)].
