# Supplementary material for: Inflammatory and Repair Pathways Induced in Human Bronchoalveolar Lavage Cells with Ozone Inhalation
Source: PLoS One. 2015 Jun 2;10(6):e0127283. doi: 10.1371/journal.pone.0127283 (PMC4452717; doi:10.1371/journal.pone.0127283)
Supplement: S1 Table — Data presented as the number of subjects in each exposure-order assignment. Pearson chi-squared p-value = 0.09. (DOCX) [file pone.0127283.s004.docx]

**S1 Table-**

| **Order of Exposures** | **0 ppb Ozone** | **100 ppb Ozone** | **200 ppb Ozone** |
| --- | --- | --- | --- |
| **First** | 7 | 6 | 6 |
| **Second** | 10 | 4 | 5 |
| **Third** | 2 | 9 | 8 |
