# Supplementary material for: Inflammatory and Repair Pathways Induced in Human Bronchoalveolar Lavage Cells with Ozone Inhalation
Source: PLoS One. 2015 Jun 2;10(6):e0127283. doi: 10.1371/journal.pone.0127283 (PMC4452717; doi:10.1371/journal.pone.0127283)
Supplement: S2 Table — Differentially expressed genes from a previous preliminary study of 3 subjects exposed to 0 and 200 ppb ozone [35]. B-score: log posterior odds ratio statistic, where odds ratio is the ratio between the probability that a given gene is differentially expressed over the probability that it is not differentially expressed (a negative B value means that the gene has a bigger chance of not being differentially expressed than being differentially expressed); FDR: false discover rate; p-values were adjusted using Holm method. Genes highlighted in bold were selected a priori for their role in migration, tissue repair and remodeling, immune response, inflammation, and extracellular region (see Methods). (DOCX) [file pone.0127283.s005.docx]

**S2 Table-**

| **Gene Symbol** | **B-Score** | **FDR** | **Adjusted p-value** |
| --- | --- | --- | --- |
| **SPP1** | 13.511 | <0.001 | 0 |
| **PRSS11** | 9.684 | <0.001 | 0 |
| TM4-B | 9.663 | <0.001 | 0 |
| CST6 | 9.643 | <0.001 | 0 |
| **CXCL11** | 9.04 | <0.001 | 0 |
| LOC129607 | 8.825 | <0.001 | 0.001 |
| SDS | 7.964 | <0.001 | 0.002 |
| **CD1C** | 7.256 | <0.001 | 0.003 |
| CACNA2D3 | 6.962 | 0.001 | 0.005 |
| **CXCL10** | 6.593 | 0.001 | 0.007 |
| NME1 | 6.518 | 0.001 | 0.008 |
| SLC6A4 | 6.335 | 0.001 | 0.01 |
| **FCN1** | 6.298 | 0.001 | 0.01 |
| **CD1B** | 6.137 | 0.001 | 0.012 |
| ADAM8 | 5.783 | 0.001 | 0.018 |
| **HAMP** | 5.713 | 0.001 | 0.02 |
| ST14 | 5.701 | 0.001 | 0.02 |
| C6orf108 | 5.599 | 0.001 | 0.022 |
| LPIN1 | 5.489 | 0.001 | 0.025 |
| PDE4B | 5.163 | 0.002 | 0.037 |
| **TIMP1** | 4.827 | 0.003 | 0.053 |
| GPR97 | 4.718 | 0.003 | 0.06 |
| POLR2J | 4.6 | 0.003 | 0.069 |
| **NR4A2** | 4.518 | 0.003 | 0.075 |
| PNG pseudogene | 4.518 | 0.003 | 0.075 |
| **PLA2G7** | 4.474 | 0.003 | 0.079 |
| AMPD3 | 4.421 | 0.003 | 0.084 |
| **COL4A6** | 4.411 | 0.003 | 0.085 |
| PP1201 | 4.364 | 0.003 | 0.089 |
| HS3ST1 | 4.304 | 0.003 | 0.096 |
| **HIST2H2BE** | 4.112 | 0.004 | 0.118 |
| **STAB1** | 3.973 | 0.004 | 0.138 |
| **CHST2** | 3.957 | 0.004 | 0.14 |
| MRC2 | 3.736 | 0.005 | 0.179 |
| **RGS1** | 3.703 | 0.005 | 0.186 |
| ALG3 | 3.557 | 0.006 | 0.219 |
| SDSL | 3.487 | 0.006 | 0.236 |
| **CD1A** | 3.46 | 0.006 | 0.243 |
| **SULF2** | 3.353 | 0.007 | 0.274 |
| ITGA2B | 3.281 | 0.007 | 0.297 |
| PER3 | 3.22 | 0.008 | 0.317 |
| AP1M2 | 3.184 | 0.008 | 0.33 |
| LRG1 | 3.127 | 0.008 | 0.352 |
| SDC4 | 2.971 | 0.009 | 0.418 |
| SPINK1 | 2.942 | 0.009 | 0.431 |
| **CSF3R** | 2.932 | 0.009 | 0.436 |
| PLXNC1 | 2.877 | 0.01 | 0.463 |
| **ARID5B** | 2.873 | 0.01 | 0.465 |
| PP3856 | 2.82 | 0.01 | 0.494 |
| **CTSK** | 2.815 | 0.01 | 0.496 |
| **HIST2H2BE** | 2.806 | 0.01 | 0.501 |
| **CCL17** | 2.794 | 0.01 | 0.508 |
| HM13 | 2.671 | 0.011 | 0.581 |
| **S100A12** | 2.607 | 0.012 | 0.624 |
| **CD36** | 2.578 | 0.012 | 0.644 |
| **CMRF-35H** | 2.56 | 0.012 | 0.657 |
| GRLF1 | 2.536 | 0.012 | 0.675 |
| **IL8RA** | 2.506 | 0.012 | 0.697 |
| **CLECSF14** | 2.504 | 0.012 | 0.699 |
| NAP1L4 | 2.452 | 0.012 | 0.74 |
| **CCR3** | 2.391 | 0.013 | 0.791 |
| GPR171 | 2.343 | 0.013 | 0.834 |
| G0S2 | 2.295 | 0.014 | 0.879 |
| **CCL2** | 2.282 | 0.014 | 0.891 |
| GADD45B | 2.127 | 0.016 | 1 |
| MINOR | 2.119 | 0.016 | 1 |
| H2AFX | 2.11 | 0.016 | 1 |
| **VCAM1** | 2.104 | 0.016 | 1 |
| BLOC1S1 | 2.057 | 0.016 | 1 |
| **GBP5** | 2.056 | 0.016 | 1 |
| DBI | 2.005 | 0.017 | 1 |
| **IL1R2** | 1.965 | 0.018 | 1 |
| MGC2731 | 1.894 | 0.019 | 1 |
| DLAT | 1.843 | 0.019 | 1 |
| MTX1 | 1.832 | 0.019 | 1 |
| FOSB | 1.773 | 0.02 | 1 |
| GTPBP6 | 1.764 | 0.02 | 1 |
| **ITK** | 1.755 | 0.02 | 1 |
| KCNJ15 | 1.752 | 0.02 | 1 |
| ACOX2 | 1.715 | 0.021 | 1 |
| **IL6** | 1.666 | 0.022 | 1 |
| TRUB2 | 1.66 | 0.022 | 1 |
| STUB1 | 1.617 | 0.022 | 1 |
| PMP22 | 1.59 | 0.022 | 1 |
| HIST2H2BE | 1.588 | 0.022 | 1 |
| DKFZP434B044 | 1.48 | 0.025 | 1 |
| CSNK1D | 1.472 | 0.025 | 1 |
| SART1 | 1.462 | 0.025 | 1 |
| PITPN | 1.361 | 0.028 | 1 |
| FYN | 1.348 | 0.028 | 1 |
| **SNCA** | 1.319 | 0.028 | 1 |
| LRP3 | 1.31 | 0.028 | 1 |
| C16orf33 | 1.295 | 0.028 | 1 |
| **MMP2** | 1.295 | 0.028 | 1 |
| OACT2 | 1.263 | 0.029 | 1 |
| SRM | 1.233 | 0.029 | 1 |
| PDIR | 1.218 | 0.029 | 1 |
| FLJ22573 | 1.216 | 0.029 | 1 |
| RPP25 | 1.171 | 0.03 | 1 |
| **PROK2** | 1.137 | 0.031 | 1 |
| NDUFS7 | 1.056 | 0.034 | 1 |
| ZNF826P | 1.055 | 0.034 | 1 |
| CDSN | 1.022 | 0.034 | 1 |
| FLJ32028 | 1.021 | 0.034 | 1 |
| SEPX1 | 1.008 | 0.034 | 1 |
| IFIX | 0.957 | 0.036 | 1 |
| UBD | 0.947 | 0.036 | 1 |
| **MMP1** | 0.923 | 0.037 | 1 |
| **LILRA3** | 0.91 | 0.037 | 1 |
| FLJ20701 | 0.835 | 0.04 | 1 |
| TDE2L | 0.805 | 0.041 | 1 |
| HIST2H2BE | 0.747 | 0.043 | 1 |
| SEC63 | 0.717 | 0.044 | 1 |
| KIAA1414 | 0.648 | 0.047 | 1 |
| CRIM1 | 0.61 | 0.048 | 1 |
| KMO | 0.61 | 0.048 | 1 |
| **S100P** | 0.607 | 0.048 | 1 |
| FLJ20045 | 0.585 | 0.048 | 1 |
| FBP1 | 0.584 | 0.048 | 1 |
| EXOSC2 | 0.559 | 0.049 | 1 |
| **CCL4** | 0.549 | 0.049 | 1 |
