# Supplementary material for: Inflammatory and Repair Pathways Induced in Human Bronchoalveolar Lavage Cells with Ozone Inhalation
Source: PLoS One. 2015 Jun 2;10(6):e0127283. doi: 10.1371/journal.pone.0127283 (PMC4452717; doi:10.1371/journal.pone.0127283)
Supplement: S3 Table — Data presented as mean±SD. a, b, c: indicate pair-wise comparisons with significant differences (p<0.05). VE: average minute ventilation during exercise; O3: ozone. (DOCX) [file pone.0127283.s006.docx]

**S3 Table-**

| **Subject Characteristics** | **0 ppb O_3_** | **100 ppb O_3_** | **200 ppb O_3_** |
| --- | --- | --- | --- |
| Temperature (°C) | 16.6±1.9 | 16.4±2.2 | 16.6±1.7 |
| Relative Humidity (%) | 53.0±9.7 | 55.9±12.2 | 50.2±8.0 |
| O_3_ concentration (ppb) | 15±5 | 107±5 | 205±7 |
| V_E_ (L/min/m^2^ BSA) | 22.1±2.2 | 22.2±3.5 | 22.0±3.4 |
