# Supplementary material for: Inflammatory and Repair Pathways Induced in Human Bronchoalveolar Lavage Cells with Ozone Inhalation
Source: PLoS One. 2015 Jun 2;10(6):e0127283. doi: 10.1371/journal.pone.0127283 (PMC4452717; doi:10.1371/journal.pone.0127283)
Supplement: S4 Table — The significant differentially expressed genes from PADE pair-wise analysis are listed with PADE delta values and fold changes. PADE Delta significance threshold for 0 versus 200 ppb pair-wise comparison = 0.7. Genes highlighted in bold represent the DEGs whose expressions were also up-regulated in the separate preliminary study of 3 subjects (see Methods and Supplemental S2 Table). (DOCX) [file pone.0127283.s007.docx]

**S4 Table-**

| **Gene Descriptor** | **Fold Change** | **PADE (Delta)** | **FDR** | **Adjusted p-value** |
| --- | --- | --- | --- | --- |
| **SPP1** | 5.73 | 2.00 | <0.001 | <0.0001 |
| **IL8RA/CXCR1/CD181** | 2.96 | 1.80 | <0.001 | <0.0001 |
| **S100A12** | 2.78 | 1.80 | <0.001 | <0.0001 |
| **IL1R2/CD121b** | 2.33 | 1.80 | <0.001 | <0.0001 |
| GPR84 | 1.81 | 1.80 | <0.001 | <0.0001 |
| **PLXNC1** | 2.36 | 1.60 | <0.001 | <0.0001 |
| **KCNJ15** | 2.12 | 1.60 | <0.001 | <0.0001 |
| PI3 | 1.91 | 1.60 | <0.001 | <0.0001 |
| **CCL2/mcp-1** | 2.90 | 1.50 | <0.001 | 0.0002 |
| Steap4 | 2.65 | 1.50 | <0.001 | 0.0001 |
| MMP9 | 1.82 | 1.50 | <0.001 | 0.0000 |
| HTRA1 | 1.37 | 1.50 | <0.001 | 0.0000 |
| IL8 | 2.22 | 1.40 | <0.001 | 0.0002 |
| **PLA2G7** | 2.59 | 1.30 | <0.001 | 0.0006 |
| MERTK | 2.26 | 1.30 | <0.001 | 0.0002 |
| CD1E | 2.23 | 1.30 | <0.001 | 0.0004 |
| **CD1C** | 2.11 | 1.30 | <0.001 | 0.0003 |
| CX3CR1 | 1.80 | 1.30 | <0.001 | 0.0002 |
| **SPINK1** | 1.66 | 1.30 | <0.001 | 0.0001 |
| SLC25A37 | 1.65 | 1.30 | <0.001 | 0.0001 |
| PID1 | 1.55 | 1.30 | <0.001 | 0.0001 |
| MMP8 | 1.50 | 1.30 | <0.001 | 0.0000 |
| MGAM | 1.49 | 1.30 | <0.001 | 0.0000 |
| **SDS** | 2.06 | 1.20 | <0.001 | 0.0006 |
| GPR183 | 2.00 | 1.20 | <0.001 | 0.0005 |
| CHST15 | 1.67 | 1.20 | <0.001 | 0.0002 |
| HGF | 1.59 | 1.20 | <0.001 | 0.0002 |
| **SULF2** | 1.57 | 1.20 | <0.001 | 0.0001 |
| RASSF2 | 2.01 | 1.10 | <0.001 | 0.0007 |
| 7896695 sequence | 1.84 | 1.10 | <0.001 | 0.0006 |
| 7896703 sequence | 1.81 | 1.10 | <0.001 | 0.0007 |
| OLFML2B | 1.69 | 1.10 | <0.001 | 0.0005 |
| 7956741 sequence | 1.52 | 1.10 | <0.001 | 0.0002 |
| **AMPD3** | 1.42 | 1.10 | <0.001 | 0.0001 |
| CRISPLD2 | 1.30 | 1.10 | <0.001 | 0.0000 |
| ANKRD22 | 1.96 | 1.00 | <0.001 | 0.0016 |
| 7896709 sequence | 1.74 | 1.00 | <0.001 | 0.0012 |
| **CD1A** | 1.72 | 1.00 | <0.001 | 0.0009 |
| ACPP | 1.70 | 1.00 | <0.001 | 0.0010 |
| LAMP3 | 1.70 | 1.00 | <0.001 | 0.0010 |
| SLC39A8 | 1.68 | 1.00 | <0.001 | 0.0010 |
| 7896727 sequence | 1.59 | 1.00 | <0.001 | 0.0007 |
| **PROK2** | 1.58 | 1.00 | <0.001 | 0.0004 |
| SLC7A5 | 1.58 | 1.00 | <0.001 | 0.0005 |
| C15orf48 | 1.57 | 1.00 | <0.001 | 0.0008 |
| MEF2C | 1.54 | 1.00 | <0.001 | 0.0005 |
| **STAB1** | 1.42 | 1.00 | <0.001 | 0.0003 |
| CCL22 | 1.89 | 0.90 | 0.014 | 0.0019 |
| SERPINB9 | 1.75 | 0.90 | 0.014 | 0.0022 |
| 7896687 sequence | 1.72 | 0.90 | 0.014 | 0.0019 |
| SELL | 1.72 | 0.90 | 0.014 | 0.0016 |
| **CD1B** | 1.71 | 0.90 | 0.014 | 0.0016 |
| PRKCB | 1.66 | 0.90 | 0.014 | 0.0013 |
| SLC7A11 | 1.58 | 0.90 | 0.014 | 0.0010 |
| ETV5 | 1.55 | 0.90 | 0.014 | 0.0011 |
| **GPR97** | 1.54 | 0.90 | 0.014 | 0.0010 |
| **CSF3R** | 1.51 | 0.90 | 0.014 | 0.0012 |
| TLR2 | 1.51 | 0.90 | 0.014 | 0.0007 |
| FAM65B | 1.51 | 0.90 | 0.014 | 0.0009 |
| CXCR4 | 1.50 | 0.90 | 0.014 | 0.0011 |
| EMR2 | 1.48 | 0.90 | 0.014 | 0.0008 |
| SLC25A37 | 1.48 | 0.90 | 0.014 | 0.0007 |
| LILRA1 | 1.46 | 0.90 | 0.014 | 0.0011 |
| 7894130 sequence | 1.45 | 0.90 | 0.014 | 0.0007 |
| LOC643332 | 1.40 | 0.90 | 0.014 | 0.0005 |
| **MMP2** | 1.36 | 0.90 | 0.014 | 0.0003 |
| LEPREL1 | 1.35 | 0.90 | 0.014 | 0.0003 |
| SIGLEC10 | 1.33 | 0.90 | 0.014 | 0.0002 |
| IFITM2 | 1.33 | 0.90 | 0.014 | 0.0003 |
| TLR10 | 1.32 | 0.90 | 0.014 | 0.0001 |
| CCR2 | 1.78 | 0.80 | 0.023 | 0.0040 |
| CCR2 | 1.78 | 0.80 | 0.023 | 0.0038 |
| C4orf18 | 1.73 | 0.80 | 0.023 | 0.0027 |
| MMP12 | 1.73 | 0.80 | 0.023 | 0.0038 |
| FCGR2B | 1.70 | 0.80 | 0.023 | 0.0037 |
| IDO1 | 1.62 | 0.80 | 0.023 | 0.0025 |
| CORO1A | 1.61 | 0.80 | 0.023 | 0.0022 |
| IL8RB | 1.53 | 0.80 | 0.023 | 0.0023 |
| HSD11B1 | 1.46 | 0.80 | 0.023 | 0.0019 |
| CLEC10A | 1.45 | 0.80 | 0.023 | 0.0012 |
| **DKFZP564O0823** | 1.44 | 0.80 | 0.023 | 0.0010 |
| MARCKS | 1.40 | 0.80 | 0.023 | 0.0008 |
| NT5DC2 | 1.36 | 0.80 | 0.023 | 0.0009 |
| PGA3 | 1.30 | 0.80 | 0.023 | 0.0004 |
| SEMA4A | 1.29 | 0.80 | 0.023 | 0.0004 |
| PLEK2 | 1.28 | 0.80 | 0.023 | 0.0002 |
| CYTSB | 1.23 | 0.80 | 0.023 | <0.0001 |
| EMP1 | 1.73 | 0.70 | 0.045 | 0.0065 |
| TNFAIP6 | 1.72 | 0.70 | 0.045 | 0.0058 |
| CLEC5A | 1.56 | 0.70 | 0.045 | 0.0032 |
| ST8SIA4 | 1.54 | 0.70 | 0.045 | 0.0030 |
| IER3 | 1.51 | 0.70 | 0.045 | 0.0034 |
| IER3 | 1.51 | 0.70 | 0.045 | 0.0034 |
| SOD2 | 1.38 | 0.70 | 0.045 | 0.0016 |
| GAL3ST4 | 1.35 | 0.70 | 0.045 | 0.0018 |
| TNFRSF10C | 1.34 | 0.70 | 0.045 | 0.0018 |
| PGA5 | 1.29 | 0.70 | 0.045 | 0.0005 |
| PLD4 | 1.27 | 0.70 | 0.045 | 0.0004 |
| ADM | 1.26 | 0.70 | 0.045 | 0.0004 |
| KCNK13 | 1.20 | 0.70 | 0.045 | 0.0001 |
