# Supplementary material for: Inflammatory and Repair Pathways Induced in Human Bronchoalveolar Lavage Cells with Ozone Inhalation
Source: PLoS One. 2015 Jun 2;10(6):e0127283. doi: 10.1371/journal.pone.0127283 (PMC4452717; doi:10.1371/journal.pone.0127283)
Supplement: S5 Table — The significant differentially expressed genes from PADE pair-wise analysis are listed with PADE delta values and fold changes. PADE Delta significance threshold for 100 versus 200 ppb pair-wise comparison = 0.5. (DOCX) [file pone.0127283.s008.docx]

**S5 Table-**

| **Gene Descriptor** | **Fold Change** | **PADE (Delta)** | **FDR** | **Adjusted p-value** |
| --- | --- | --- | --- | --- |
| SPPI | 2.68 | 0.60 | 0.042 | 0.0072 |
| CCL2 | 2.31 | 0.60 | 0.042 | 0.0038 |
| S100A12 | 2.22 | 0.60 | 0.042 | 0.0007 |
| IL8RA | 2.21 | 0.60 | 0.042 | 0.0015 |
| PLXNC1 | 1.98 | 0.60 | 0.042 | 0.0009 |
| MERTK | 1.93 | 0.60 | 0.042 | 0.0015 |
| RASSF2 | 1.85 | 0.60 | 0.042 | 0.0006 |
| CCR2 | 1.79 | 0.60 | 0.042 | 0.0032 |
| IL1R2 | 1.77 | 0.60 | 0.042 | 0.0011 |
| ANKRD22 | 1.75 | 0.60 | 0.042 | 0.0030 |
| 7896687 sequence | 1.73 | 0.60 | 0.042 | 0.0029 |
| CD1C | 1.70 | 0.60 | 0.042 | 0.0016 |
| SELL | 1.70 | 0.60 | 0.042 | 0.0020 |
| CORO1A | 1.69 | 0.60 | 0.042 | 0.0026 |
| 7896703 sequence | 1.66 | 0.60 | 0.042 | 0.0024 |
| KCNJ15 | 1.65 | 0.60 | 0.042 | 0.0022 |
| ACPP | 1.61 | 0.60 | 0.042 | 0.0019 |
| SLC25A37 | 1.60 | 0.60 | 0.042 | 0.0013 |
| MEF2C | 1.59 | 0.60 | 0.042 | 0.0007 |
| GPR84 | 1.56 | 0.60 | 0.042 | 0.0006 |
| FAM65B | 1.53 | 0.60 | 0.042 | 0.0006 |
| 7896727 sequence | 1.52 | 0.60 | 0.042 | 0.0004 |
| SLC25A37 | 1.49 | 0.60 | 0.042 | 0.0009 |
| GPR141 | 1.49 | 0.60 | 0.042 | 0.0003 |
| 7892990 sequence | 1.95 | 0.50 | 0.071 | 0.0095 |
| STEAP4 | 1.89 | 0.50 | 0.071 | 0.0049 |
| TMEM154 | 1.72 | 0.50 | 0.071 | 0.0065 |
| CD180 | 1.71 | 0.50 | 0.071 | 0.0065 |
| C4orf18 | 1.70 | 0.50 | 0.071 | 0.0034 |
| CD1E | 1.70 | 0.50 | 0.071 | 0.0043 |
| GPR183 | 1.69 | 0.50 | 0.071 | 0.0045 |
| SERPINB9 | 1.67 | 0.50 | 0.071 | 0.0066 |
| CLEC5A | 1.60 | 0.50 | 0.071 | 0.0050 |
| ST8SIA4 | 1.55 | 0.50 | 0.071 | 0.0023 |
| IER3 | 1.50 | 0.50 | 0.071 | 0.0021 |
| IER3 | 1.50 | 0.50 | 0.071 | 0.0021 |
| IER3 | 1.49 | 0.50 | 0.071 | 0.0028 |
| ICAM3 | 1.48 | 0.50 | 0.071 | 0.0020 |
| LILRA1 | 1.47 | 0.50 | 0.071 | 0.0012 |
| HGF | 1.47 | 0.50 | 0.071 | 0.0015 |
| SULF2 | 1.42 | 0.50 | 0.071 | 0.0014 |
| IFITM2 | 1.37 | 0.50 | 0.071 | 0.0007 |
