# Supplementary material for: Inflammatory and Repair Pathways Induced in Human Bronchoalveolar Lavage Cells with Ozone Inhalation
Source: PLoS One. 2015 Jun 2;10(6):e0127283. doi: 10.1371/journal.pone.0127283 (PMC4452717; doi:10.1371/journal.pone.0127283)
Supplement: S6 Table — GSA identified several processes associated with the DEGs from linear regression analysis with FDR threshold <25%. (DOCX) [file pone.0127283.s009.docx]

**S6 Table-**

| **Set Name** | **GSA Score** | **GSA**  **p-value** | **GSA FDR (%)** |
| --- | --- | --- | --- |
| KEGG_HEMATOPOIETIC_CELL_LINEAGE | 0.6339 | <0.001 | <0.1 |
| BIOCARTA_CALCINEURIN_PATHWAY | 0.688 | <0.001 | <0.1 |
| BIOCARTA_CCR5_PATHWAY | 0.8573 | <0.001 | <0.1 |
| KEGG_CYTOKINE_CYTOKINE_RECEPTOR_INTERACTION | 0.3848 | <0.001 | <0.1 |
| G_PROTEIN_COUPLED_RECEPTOR_BINDING | 0.6667 | <0.001 | <0.1 |
| METALLOPEPTIDASE_ACTIVITY | 0.4574 | <0.001 | <0.1 |
| CHEMOKINE_ACTIVITY | 0.684 | <0.001 | <0.1 |
| CHEMOKINE_RECEPTOR_BINDING | 0.787 | <0.001 | <0.1 |
| METALLOENDOPEPTIDASE_ACTIVITY | 0.6465 | <0.001 | <0.1 |
| BEHAVIOR | 0.4988 | <0.001 | <0.1 |
| LOCOMOTORY_BEHAVIOR | 0.6903 | <0.001 | <0.1 |
| INFLAMMATORY_RESPONSE | 0.4218 | <0.001 | <0.1 |
| RESPONSE_TO_EXTERNAL_STIMULUS | 0.3522 | <0.001 | <0.1 |
| RESPONSE_TO_WOUNDING | 0.4154 | <0.001 | <0.1 |
| KEGG_ALDOSTERONE_REGULATED_SODIUM_REABSORPTION | 0.4825 | 0.002 | 4.5 |
| BIOCARTA_CARDIACEGF_PATHWAY | 0.6509 | 0.002 | 4.5 |
| KEGG_CHEMOKINE_SIGNALING_PATHWAY | 0.2868 | 0.002 | 4.5 |
| AMINO_ACID_TRANSMEMBRANE_TRANSPORTER_ACTIVITY | 0.5571 | 0.002 | 6.4 |
| ENDOPEPTIDASE_ACTIVITY | 0.2412 | 0.002 | 6.4 |
| RECEPTOR_BINDING | 0.1803 | 0.002 | 6.4 |
| CYTOKINE_ACTIVITY | 0.3227 | 0.002 | 6.4 |
| SIGNAL_TRANSDUCTION | 0.1081 | 0.002 | 6.4 |
| DEFENSE_RESPONSE | 0.3444 | 0.002 | 6.4 |
| RESPONSE_TO_OTHER_ORGANISM | 0.3027 | 0.002 | 6.4 |
| SUGAR_BINDING | 0.4906 | 0.004 | 9.4 |
| ORGANIC_ACID_TRANSMEMBRANE_TRANSPORTER_ACTIVITY | 0.4135 | 0.004 | 9.4 |
| CARBOXYLIC_ACID_TRANSMEMBRANE_TRANSPORTER_ACTIVITY | 0.4289 | 0.004 | 9.4 |
| CELL_SURFACE_RECEPTOR_LINKED_SIGNAL_TRANSDUCTION_GO_0007166 | 0.1863 | 0.004 | 9.4 |
| POSITIVE_REGULATION_OF_CASPASE_ACTIVITY | 0.4208 | 0.004 | 9.4 |
| CYTOKINE_SECRETION | 0.6489 | 0.004 | 9.4 |
| KEGG_AXON_GUIDANCE | 0.3004 | 0.006 | 9.5 |
| KEGG_BLADDER_CANCER | 0.3378 | 0.006 | 9.5 |
| BIOCARTA_SPPA_PATHWAY | 0.5623 | 0.006 | 9.5 |
| INTEGRAL_TO_PLASMA_MEMBRANE | 0.1749 | 0.006 | 12.5 |
| INTRINSIC_TO_PLASMA_MEMBRANE | 0.1716 | 0.006 | 12.5 |
| MULTI_ORGANISM_PROCESS | 0.1734 | 0.006 | 12.5 |
| BIOCARTA_PGC1A_PATHWAY | 0.4953 | 0.01 | 14.4 |
| CARBOHYDRATE_BINDING | 0.2929 | 0.008 | 15.5 |
| ENZYME_INHIBITOR_ACTIVITY | 0.2252 | 0.01 | 15.5 |
| RECEPTOR_ACTIVITY | 0.1777 | 0.01 | 15.5 |
| PATTERN_BINDING | 0.3259 | 0.008 | 15.5 |
| EXTRACELLULAR_SPACE | 0.2427 | 0.01 | 15.5 |
| NEGATIVE_REGULATION_OF_TRANSPORT | 0.5366 | 0.01 | 15.5 |
| REGULATION_OF_CYTOKINE_SECRETION | 0.6891 | 0.01 | 15.5 |
| NEGATIVE_REGULATION_OF_CELL_CYCLE | 0.2243 | 0.01 | 15.5 |
| REGULATION_OF_PROTEIN_IMPORT_INTO_NUCLEUS | 0.6613 | 0.01 | 15.5 |
| KEGG_RENIN_ANGIOTENSIN_SYSTEM | 0.616 | 0.01 | 15.9 |
| BIOCARTA_CXCR4_PATHWAY | 0.4247 | 0.01 | 15.9 |
| KEGG_GLYCOSAMINOGLYCAN_BIOSYNTHESIS_HEPARAN_SULFATE | 0.4588 | 0.01 | 15.9 |
| PROTEASE_INHIBITOR_ACTIVITY | 0.3768 | 0.01 | 16.7 |
| RESPONSE_TO_CHEMICAL_STIMULUS | 0.1584 | 0.01 | 16.7 |
| BONE_REMODELING | 0.4251 | 0.01 | 16.7 |
| CATION_HOMEOSTASIS | 0.2759 | 0.01 | 16.7 |
| L_AMINO_ACID_TRANSMEMBRANE_TRANSPORTER_ACTIVITY | 0.6197 | 0.01 | 17.7 |
| TRANSFERASE_ACTIVITY_TRANSFERRING_SULFUR_CONTAINING_GROUPS | 0.4578 | 0.01 | 17.7 |
| REGULATION_OF_DEVELOPMENTAL_PROCESS | 0.1158 | 0.01 | 17.7 |
| DEFENSE_RESPONSE_TO_BACTERIUM | 0.4456 | 0.01 | 17.7 |
| EXTRACELLULAR_REGION_PART | 0.2261 | 0.01 | 18.5 |
| REGULATION_OF_NUCLEOCYTOPLASMIC_TRANSPORT | 0.4911 | 0.01 | 18.5 |
| CELLULAR_CATION_HOMEOSTASIS | 0.2821 | 0.01 | 18.5 |
| G_PROTEIN_COUPLED_RECEPTOR_PROTEIN_SIGNALING_PATHWAY | 0.2598 | 0.01 | 18.5 |
| ZINC_ION_BINDING | 0.2468 | 0.02 | 18.7 |
| PLASMA_MEMBRANE | 0.1338 | 0.02 | 18.7 |
| REGULATION_OF_SECRETION | 0.3715 | 0.01 | 18.7 |
| AMINO_ACID_TRANSPORT | 0.3462 | 0.01 | 18.7 |
| EXTRACELLULAR_REGION | 0.2007 | 0.01 | 18.7 |
| PROTEIN_SECRETION | 0.3363 | 0.02 | 18.7 |
| VASCULATURE_DEVELOPMENT | 0.3455 | 0.02 | 18.7 |
| CELLULAR_HOMEOSTASIS | 0.2037 | 0.02 | 18.7 |
| ION_HOMEOSTASIS | 0.2198 | 0.01 | 18.7 |
| TISSUE_REMODELING | 0.36 | 0.02 | 18.7 |
| POSITIVE_REGULATION_OF_SECRETION | 0.5208 | 0.01 | 18.7 |
| CELLULAR_DEFENSE_RESPONSE | 0.3424 | 0.02 | 18.8 |
| SULFOTRANSFERASE_ACTIVITY | 0.5174 | 0.02 | 18.8 |
| LIPOPROTEIN_BINDING | 0.3631 | 0.02 | 18.8 |
| INTEGRAL_TO_MEMBRANE | 0.1114 | 0.02 | 18.8 |
| AMINE_TRANSMEMBRANE_TRANSPORTER_ACTIVITY | 0.3782 | 0.02 | 18.8 |
| PLASMA_MEMBRANE_PART | 0.1447 | 0.02 | 18.8 |
| JAK_STAT_CASCADE | 0.3683 | 0.02 | 18.8 |
| REGULATION_OF_MOLECULAR_FUNCTION | 0.097 | 0.02 | 18.8 |
| CYTOKINE_PRODUCTION | 0.2792 | 0.02 | 18.8 |
| POSITIVE_REGULATION_OF_DEVELOPMENTAL_PROCESS | 0.1125 | 0.02 | 18.8 |
| REGULATION_OF_CATALYTIC_ACTIVITY | 0.1247 | 0.02 | 18.8 |
| SECOND_MESSENGER_MEDIATED_SIGNALING | 0.2573 | 0.02 | 18.8 |
| REGULATION_OF_INTRACELLULAR_TRANSPORT | 0.4002 | 0.02 | 18.8 |
| POSITIVE_REGULATION_OF_BIOLOGICAL_PROCESS | 0.0769 | 0.02 | 18.8 |
| NEGATIVE_REGULATION_OF_CATALYTIC_ACTIVITY | 0.2472 | 0.02 | 18.8 |
| NEGATIVE_REGULATION_OF_DEVELOPMENTAL_PROCESS | 0.1537 | 0.02 | 18.8 |
| REGULATION_OF_PROTEIN_SECRETION | 0.4868 | 0.02 | 18.8 |
| ANTIGEN_BINDING | 0.5891 | 0.02 | 19.8 |
| REGULATION_OF_HEART_CONTRACTION | 0.4873 | 0.02 | 19.8 |
| POSITIVE_REGULATION_OF_CELLULAR_PROTEIN_METABOLIC_PROCESS | 0.2606 | 0.03 | 20.9 |
| BIOCARTA_AT1R_PATHWAY | 0.3999 | 0.02 | 21.2 |
| CELL_DEVELOPMENT | 0.0731 | 0.03 | 21.5 |
| POSITIVE_REGULATION_OF_CELLULAR_PROCESS | 0.0678 | 0.03 | 21.5 |
| REGULATION_OF_MULTICELLULAR_ORGANISMAL_PROCESS | 0.2235 | 0.03 | 21.5 |
| BIOCARTA_EDG1_PATHWAY | 0.37 | 0.02 | 21.8 |
| INORGANIC_ANION_TRANSMEMBRANE_TRANSPORTER_ACTIVITY | 0.5359 | 0.03 | 22.0 |
| POSITIVE_REGULATION_OF_PROTEIN_METABOLIC_PROCESS | 0.2438 | 0.03 | 22.0 |
| NEGATIVE_REGULATION_OF_TRANSFERASE_ACTIVITY | 0.3503 | 0.03 | 22.0 |
| POTASSIUM_CHANNEL_ACTIVITY | 0.3913 | 0.03 | 22.7 |
| TRANSMEMBRANE_RECEPTOR_ACTIVITY | 0.1664 | 0.03 | 22.7 |
| REGULATION_OF_BIOLOGICAL_QUALITY | 0.0881 | 0.03 | 22.7 |
| MICROTUBULE_ORGANIZING_CENTER_PART | 0.4637 | 0.03 | 22.7 |
| NEGATIVE_REGULATION_OF_CELL_DIFFERENTIATION | 0.4158 | 0.03 | 22.7 |
| REGULATION_OF_TRANSPORT | 0.2002 | 0.03 | 22.7 |
| POTASSIUM_ION_TRANSPORT | 0.3328 | 0.03 | 22.7 |
| PROTEIN_KINASE_INHIBITOR_ACTIVITY | 0.3883 | 0.04 | 22.9 |
| INTRINSIC_TO_MEMBRANE | 0.107 | 0.04 | 22.9 |
| METAL_ION_TRANSPORT | 0.2208 | 0.04 | 22.9 |
| REGULATION_OF_MITOTIC_CELL_CYCLE | 0.3564 | 0.04 | 22.9 |
| KINASE_INHIBITOR_ACTIVITY | 0.3939 | 0.04 | 23.0 |
| REGULATION_OF_KINASE_ACTIVITY | 0.1262 | 0.04 | 23.0 |
| REGULATION_OF_PROTEIN_KINASE_ACTIVITY | 0.1232 | 0.04 | 23.0 |
| REGULATION_OF_TRANSFERASE_ACTIVITY | 0.1178 | 0.04 | 23.0 |
| DETECTION_OF_STIMULUS | 0.3334 | 0.04 | 23.0 |
| MYELOID_LEUKOCYTE_DIFFERENTIATION | 0.4524 | 0.04 | 23.0 |
| HOMEOSTATIC_PROCESS | 0.1413 | 0.04 | 23.0 |
| IMMUNE_SYSTEM_PROCESS | 0.2006 | 0.04 | 23.0 |
| POSITIVE_REGULATION_OF_CELL_CYCLE | 0.5064 | 0.04 | 23.0 |
| NEGATIVE_REGULATION_OF_DNA_METABOLIC_PROCESS | 0.3932 | 0.04 | 23.2 |
| POSITIVE_REGULATION_OF_CATALYTIC_ACTIVITY | 0.1166 | 0.04 | 23.2 |
| CHEMICAL_HOMEOSTASIS | 0.1625 | 0.04 | 23.2 |
| REGULATION_OF_APOPTOSIS | 0.1222 | 0.04 | 23.2 |
| SECONDARY_ACTIVE_TRANSMEMBRANE_TRANSPORTER_ACTIVITY | 0.2661 | 0.04 | 23.5 |
| BASEMENT_MEMBRANE | 0.3488 | 0.04 | 23.5 |
| REGULATION_OF_PROGRAMMED_CELL_DEATH | 0.1208 | 0.04 | 23.5 |
| INTERLEUKIN_BINDING | 0.4163 | 0.05 | 23.8 |
| RECEPTOR_COMPLEX | 0.241 | 0.05 | 23.8 |
| POSITIVE_REGULATION_OF_TRANSLATION | 0.3153 | 0.05 | 23.8 |
| POSITIVE_REGULATION_OF_PHOSPHORYLATION | 0.3317 | 0.05 | 23.8 |
| REGULATION_OF_PHOSPHORYLATION | 0.2587 | 0.05 | 23.8 |
| BLOOD_COAGULATION | 0.2261 | 0.05 | 23.8 |
| RESPONSE_TO_BACTERIUM | 0.3008 | 0.05 | 23.8 |
| ANGIOGENESIS | 0.2654 | 0.05 | 23.8 |
| BIOCARTA_VEGF_PATHWAY | 0.3661 | 0.02 | 24.3 |
| MYELOID_CELL_DIFFERENTIATION | 0.3137 | 0.05 | 24.3 |
| NEGATIVE_REGULATION_OF_BIOLOGICAL_PROCESS | 0.0587 | 0.05 | 24.3 |
| POSITIVE_REGULATION_OF_RESPONSE_TO_STIMULUS | 0.2233 | 0.05 | 24.8 |
| IMMUNE_RESPONSE | 0.2133 | 0.05 | 24.8 |
| REPRODUCTION | 0.1061 | 0.05 | 24.8 |
| ION_BINDING | 0.0824 | 0.05 | 24.8 |
| POSITIVE_REGULATION_OF_CYTOKINE_BIOSYNTHETIC_PROCESS | 0.3738 | 0.05 | 24.8 |
| REGULATION_OF_BODY_FLUID_LEVELS | 0.1891 | 0.05 | 24.8 |
