# Supplementary material for: Inflammatory and Repair Pathways Induced in Human Bronchoalveolar Lavage Cells with Ozone Inhalation
Source: PLoS One. 2015 Jun 2;10(6):e0127283. doi: 10.1371/journal.pone.0127283 (PMC4452717; doi:10.1371/journal.pone.0127283)
Supplement: S7 Table — iReport generated 19 pathways that were associated with the 49 DEGs from the two-group comparison of 0 to 200 ppb ozone exposure. Pathways with a p-value < 0.01 are shown. (DOCX) [file pone.0127283.s010.docx]

**S7 Table-**

| **Pathways** | **DEGs** | **p-value** | **Genes** |
| --- | --- | --- | --- |
| Granulocyte Adhesion and Diapedesis | 8 | 1.9x10^-8^ | CCL2, IL1R2, IL8, CCL22, MMP12, MMP9, SELL, CXCL9 |
| Agranulocyte Adhesion and Diapedesis | 8 | 3.1 x10^-8^ | IL8, SELL, CXCL9, CCL2, CCL22, CXCR1, MMP12, MMP9 |
| Hepatic Fibrosis / Hepatic Stellate Cell Activation | 6 | 1.9 x10^-6^ | IL1R2, IL8, CXCL9, CCL2, HGF, MMP9 |
| Atherosclerosis Signaling | 5 | 1.8 x10^-5^ | IL8, CCL2, CCR2, PLA2G7, MMP9 |
| Lipid Antigen Presentation by CD1 | 3 | 4.5 x10^-5^ | CD1A, CD1B, CD1C |
| Airway Pathology in Chronic Obstructive Pulmonary Disease | 2 | 1.9 x10^-4^ | IL8, MMP9 |
| TREM1 Signaling | 3 | 5.3 x10^-4^ | IL8, CCL2, FCGR2B |
| Dendritic Cell Maturation | 4 | 1.3 x10^-3^ | CD1A, CD1B, CD1C, FCGR2B |
| IL-8 Signaling | 4 | 1.6 x10^-3^ | IL8, CXCR1, MMP9, PRKCB |
| Bladder Cancer Signaling | 3 | 1.7 x10^-3^ | IL8, MMP12, MMP9 |
| LXR/RXR Activation | 3 | 4.2 x10^-3^ | IL1R2, CCL2, MMP9 |
| Inhibition of Matrix Metalloproteases | 2 | 4.9 x10^-3^ | MMP12, MMP9 |
| Hepatic Cholestasis | 3 | 6.2 x10^-3^ | IL1R2, IL8, PRKCB |
| Role of Hypercytokinemia/hyperchemokinemia in the Pathogenesis of Influenza | 2 | 6.2 x10^-3^ | IL8, CCL2 |
| MSP-RON Signaling Pathway | 2 | 6.5 x10^-3^ | CCL2, CCR2 |
| Role of IL-17F in Allergic Inflammatory Airway Diseases | 2 | 6.5 x10^-3^ | IL8, CCL2 |
| L-serine Degradation | 1 | 8.0 x10^-3^ | SDS |
| Role of Macrophages, Fibroblasts and Endothelial Cells in Rheumatoid Arthritis | 4 | 9.0 x10^-3^ | IL1R2, IL8, CCL2, PRKCB |
| Role of IL-17A in Arthritis | 2 | 9.3 x10^-3^ | IL8, CCL2 |
