# Supplementary material for: Inflammatory and Repair Pathways Induced in Human Bronchoalveolar Lavage Cells with Ozone Inhalation
Source: PLoS One. 2015 Jun 2;10(6):e0127283. doi: 10.1371/journal.pone.0127283 (PMC4452717; doi:10.1371/journal.pone.0127283)
Supplement: S8 Table — iReport generated 59 processes that were associated with the 49 DEGs from the two-group comparison of 0 to 200 ppb ozone exposure. Pathways with a p-value <1x10^-8^ are shown. (DOCX) [file pone.0127283.s011.docx]

**S8 Table-**

| **Biological Process** | **DEGs** | **p-value** | **Genes** |
| --- | --- | --- | --- |
| Cell movement of leukocytes | 22 | 3.8x10^-16^ | SPP1, CCL2, PLA2G7, CXCR1, S100A12, PLXNC1, GPR183, IL8, CCL22, CCR2, CX3CR1, MMP12, MMP9, FCGR2B, SELL, PRKCB, CORO1A, IDO1, CLEC5A, CXCL9, HGF, CD207 |
| Leukocyte migration | 23 | 5.5x10^-16^ | SPP1, CCL2, PLA2G7, CXCR1, S100A12, PLXNC1, GPR183, IL8, CCL22, CCR2, CX3CR1, MMP12, MMP9, FCGR2B, SELL, PRKCB, F13A1, CORO1A, IDO1, CLEC5A, CXCL9, HGF, CD207 |
| Cell movement of monocytes | 13 | 6.4 x10^-16^ | SPP1, CCL2, PLA2G7, CXCR1, S100A12, IL8, CCL22, CCR2, MMP12, MMP9, PRKCB, CXCL9, HGF |
| Activation of blood cells | 21 | 3.5 x10^-15^ | SPP1, CCL2, S100A12, CD1E, MERTK, CD1C, IL8, CCL22, CCR2, SERPINB9, CD1A, CX3CR1, MMP9, FCGR2B, CD1B, SELL, PRKCB, CORO1A, IDO1, CLEC5A, CXCL9 |
| Activation of leukocytes | 20 | 4.7 x10^-15^ | IL8, SELL, SPP1, CXCL9, CD1B, CCL22, S100A12, CD1E, CLEC5A, FCGR2B, SERPINB9, CCL2, MERTK, CD1A, CORO1A, IDO1, CD1C, CCR2, MMP9, PRKCB |
| Activation of cells | 23 | 7.1 x10^-15^ | SPP1, CCL2, CXCR1, S100A12, CD1E, MERTK, CD1C, IL8, CCL22, CCR2, SERPINB9, CD1A, CX3CR1, MMP9, FCGR2B, CD1B, SELL, PRKCB, CORO1A, IDO1, CLEC5A, CXCL9, HGF |
| Cell movement of phagocytes | 18 | 2.2 x10^-14^ | SPP1, CCL2, PLA2G7, CXCR1, S100A12, PLXNC1, IL8, CCL22, CCR2, CX3CR1, MMP12, MMP9, SELL, PRKCB, CORO1A, CXCL9, HGF, CD207 |
| Cell movement of mononuclear leukocytes | 16 | 4.1 x10^-13^ | IL8, SELL, SPP1, CXCL9, CCL22, S100A12, CXCR1, CX3CR1, PLA2G7, GPR183, CCL2, HGF, CCR2, MMP12, MMP9, PRKCB |
| Homing of mononuclear leukocytes | 12 | 5.6 x10^-13^ | IL8, SELL, CXCL9, SPP1, CCL2, CCL22, CXCR1, S100A12, CCR2, PLA2G7, MMP9, PRKCB |
| Activation of mononuclear leukocytes | 16 | 1.1 x10^-12^ | IL8, SPP1, CD1B, CCL22, S100A12, CD1E, CLEC5A, FCGR2B, SERPINB9, CCL2, MERTK, CD1A, CORO1A, CD1C, CCR2, PRKCB |
| Chemotaxis of myeloid cells | 13 | 1.6 x10^-12^ | IL8, SELL, SPP1, CXCL9, CCL22, S100A12, CXCR1, CX3CR1, PLA2G7, CCL2, CORO1A, CCR2, PRKCB |
| Chemotaxis of leukocytes | 14 | 1.7 x10^-12^ | IL8, SELL, SPP1, CXCL9, CCL22, S100A12, CXCR1, CX3CR1, PLA2G7, CCL2, CORO1A, CCR2, MMP9, PRKCB |
| Chemotaxis of phagocytes | 13 | 2.5 x10^-12^ | IL8, SELL, SPP1, CXCL9, CCL22, S100A12, CXCR1, CX3CR1, PLA2G7, CCL2, CORO1A, CCR2, PRKCB |
| Chemotaxis of mononuclear leukocytes | 11 | 3.1 x10^-12^ | IL8, CXCL9, SPP1, CCL2, CCL22, CXCR1, S100A12, CCR2, PLA2G7, MMP9, PRKCB |
| Cell movement of myeloid cells | 16 | 3.4 x10^-12^ | IL8, SELL, SPP1, CXCL9, CCL22, S100A12, CXCR1, CX3CR1, PLA2G7, CCL2, HGF, CORO1A, CCR2, MMP12, MMP9, PRKCB |
| Inflammatory response | 18 | 3.5 x10^-12^ | IL8, SELL, SPP1, CXCL9, CCL22, S100A12, CXCR1, CLEC5A, CX3CR1, FCGR2B, PLA2G7, CCL2, HGF, CORO1A, IDO1, CCR2, MMP9, PRKCB |
| Chemotaxis of monocytes | 9 | 4.5 x10^-12^ | IL8, CXCL9, CCL2, CCL22, CXCR1, S100A12, CCR2, PLA2G7, PRKCB |
| Quantity of antigen presenting cells | 12 | 9.6 x10^-12^ | PI3, SELL, SPP1, CCL2, IDO1, CCL22, CX3CR1, MMP12, FCGR2B, CCR2, MMP9, STEAP4 |
| Migration of cells | 26 | 1.0 x10^-11^ | SPP1, CCL2, PLA2G7, CXCR1, S100A12, PLXNC1, MERTK, GPR183, IL8, CCL22, CCR2, CX3CR1, MMP12, MMP9, FCGR2B, SELL, PRKCB, F13A1, CORO1A, IDO1, CLEC5A, CXCL9, ETV5, ST8SIA4, HGF, CD207 |
| Cell movement of antigen presenting cells | 13 | 1.6 x10^-11^ | IL8, SELL, CD207, PLXNC1, SPP1, CXCL9, CCL22, CXCR1, CX3CR1, CCL2, HGF, CCR2, MMP12 |
| Migration of phagocytes | 12 | 1.8 x10^-11^ | IL8, CD207, SELL, CXCL9, SPP1, PLXNC1, CCL2, HGF, CCL22, MMP12, CCR2, MMP9 |
| Chemotaxis of cells | 15 | 2.8 x10^-11^ | IL8, SELL, SPP1, CXCL9, CCL22, S100A12, CXCR1, CX3CR1, PLA2G7, CCL2, HGF, CORO1A, CCR2, MMP9, PRKCB |
| Quantity of leukocytes | 19 | 3.6 x10^-11^ | IL8, SELL, SPP1, CCL22, CX3CR1, FCGR2B, GPR183, PI3, CCL2, MERTK, HGF, RASSF2, CORO1A, IDO1, CCR2, MMP12, MMP9, PRKCB, STEAP4 |
| Migration of antigen presenting cells | 10 | 3.7 x10^-11^ | IL8, CD207, CXCL9, SPP1, PLXNC1, CCL2, HGF, CCL22, MMP12, CCR2 |
| Cell movement of granulocytes | 13 | 5.4 x10^-11^ | IL8, SELL, SPP1, CXCL9, S100A12, CXCR1, PLA2G7, CCL2, HGF, CORO1A, CCR2, MMP12, MMP9 |
| Infiltration of leukocytes | 13 | 6.9 x10^-11^ | IL8, SELL, SPP1, CCL22, CLEC5A, FCGR2B, PLA2G7, CCL2, HGF, IDO1, CCR2, MMP12, MMP9 |
| Function of leukocytes | 15 | 1.1 x10^-10^ | IL8, SELL, SPP1, GPR84, CX3CR1, FCGR2B, GPR183, SERPINB9, CCL2, MERTK, HGF, IDO1, CCR2, MMP12, MMP9 |
| Activation of lymphocytes | 14 | 1.1 x10^-10^ | IL8, SPP1, CD1B, CCL22, CD1E, FCGR2B, SERPINB9, CCL2, MERTK, CD1A, CORO1A, CCR2, CD1C, PRKCB |
| Quantity of phagocytes | 13 | 2.5 x10^-10^ | IL8, SELL, SPP1, CCL22, CX3CR1, FCGR2B, PI3, CCL2, IDO1, CCR2, MMP12, MMP9, STEAP4 |
| Activation of myeloid cells | 11 | 2.6 x10^-10^ | IL8, SELL, CXCL9, SPP1, CCL2, CCL22, CLEC5A, S100A12, FCGR2B, CCR2, MMP9 |
| Recruitment of cells | 12 | 3.3 x10^-10^ | IL8, SELL, CXCL9, SPP1, CCL2, HGF, F13A1, CCL22, CX3CR1, FCGR2B, CCR2, MMP9 |
| Cell movement of dendritic cells | 9 | 4.5 x10^-10^ | IL8, CD207, CXCL9, SPP1, PLXNC1, CCL2, CCL22, CXCR1, CCR2 |
| Cell movement of natural killer cells | 7 | 5.0 x10^-10^ | IL8, SELL, CXCL9, SPP1, CCL2, CCL22, CX3CR1 |
| Cell movement of neutrophils | 11 | 6.8 x10^-10^ | IL8, SELL, SPP1, CCL2, HGF, CORO1A, CXCR1, S100A12, MMP12, CCR2, MMP9 |
| Quantity of dendritic cells | 8 | 7.8 x10^-10^ | PI3, SELL, SPP1, CCL2, IDO1, FCGR2B, CCR2, MMP9 |
| Recruitment of phagocytes | 10 | 1.0 x10^-9^ | IL8, SELL, SPP1, CCL2, F13A1, CCL22, CX3CR1, FCGR2B, CCR2, MMP9 |
| Cell movement of hematopoietic progenitor cells | 7 | 1.2 x10^-9^ | IL8, CXCL9, CCL2, HGF, CCL22, CCR2, MMP9 |
| Recruitment of leukocytes | 11 | 1.5 x10^-9^ | IL8, SELL, CXCL9, SPP1, CCL2, F13A1, CCL22, CX3CR1, FCGR2B, CCR2, MMP9 |
| NK cell migration | 6 | 1.5 x10^-9^ | IL8, SELL, CXCL9, CCL2, CCL22, CX3CR1 |
| Presentation of lipid | 4 | 1.6 x10^-9^ | CD1A, CD1B, CD1E, CD1C |
| Activation of phagocytes | 11 | 1.7 x10^-9^ | IL8, SELL, SPP1, MERTK, CCL2, IDO1, CCL22, CLEC5A, S100A12, CCR2, MMP9 |
| Proliferation of immune cells | 16 | 1.9 x10^-9^ | IL8, SELL, SPP1, FCGR2B, GPR183, SERPINB9, CCL2, MERTK, HGF, CORO1A, IDO1, MEF2C, CCR2, MMP9, PRKCB, ACPP |
| Function of phagocytes | 11 | 2.0 x10^-9^ | IL8, SELL, SPP1, MERTK, CCL2, HGF, IDO1, MMP12, FCGR2B, CCR2, MMP9 |
| Function of antigen presenting cells | 10 | 2.5 x10^-9^ | SELL, SPP1, MERTK, CCL2, HGF, IDO1, MMP12, FCGR2B, CCR2, MMP9 |
| Cell movement of macrophages | 10 | 2.7 x10^-9^ | IL8, SELL, CXCL9, SPP1, CCL2, HGF, CCL22, CX3CR1, MMP12, CCR2 |
| Quantity of macrophages | 9 | 3.1 x10^-9^ | PI3, SPP1, CCL2, CCL22, CX3CR1, MMP12, CCR2, MMP9, STEAP4 |
| Recruitment of lymphocytes | 7 | 3.8 x10^-9^ | IL8, SELL, CXCL9, CCL2, CCL22, CX3CR1, CCR2 |
| Recruitment of macrophages | 7 | 5.6 x10^-9^ | IL8, SPP1, CCL2, CCL22, CX3CR1, CCR2, MMP9 |
| Recruitment of granulocytes | 9 | 6.1 x10^-9^ | IL8, SELL, CXCL9, CCL2, CCL22, CX3CR1, FCGR2B, CCR2, MMP9 |
| Transmigration of cells | 8 | 6.3 x10^-9^ | IL8, SELL, CXCL9, CCL2, CCL22, CX3CR1, CCR2, MMP9 |
| Response of phagocytes | 9 | 6.3 x10^-9^ | IL8, SERPINB9, CCL2, HGF, CORO1A, CCL22, CLEC5A, FCGR2B, CCR2 |
| Infiltration of myeloid cells | 9 | 6.3 x10^-9^ | IL8, SELL, SPP1, CCL2, HGF, MMP12, CCR2, PLA2G7, MMP9 |
| Adhesion of mononuclear leukocytes | 8 | 6.6 x10^-9^ | IL8, SELL, CXCL9, SPP1, CCL2, HGF, CCL22, CXCR1 |
| Chemotaxis of antigen presenting cells | 8 | 7.4 x10^-9^ | SELL, CXCL9, SPP1, CCL2, CCL22, CX3CR1, CXCR1, CCR2 |
| Cell movement of lymphoma cell lines | 6 | 8.1 x10^-9^ | IL8, SELL, CCL2, HGF, CCL22, CXCR1 |
| Migration of mononuclear leukocytes | 11 | 9.2 x10^-9^ | GPR183, IL8, SELL, CXCL9, SPP1, CCL2, HGF, CCL22, CX3CR1, CCR2, MMP9 |
| Shape change of blood cells | 7 | 9.3 x10^-9^ | IL8, SELL, PLXNC1, CCL2, CORO1A, CCL22, CCR2 |

**Supplemental Table S9- Diseases Associated with the DEGs by iReport**. iReport generated 25 diseases that were associated with the 49 DEGs from the two-group comparison of 0 to 200 ppb ozone exposure. Diseases with a p-value <1x10^-6^ are shown.

| **Disease** | **DEGs** | **p-value** | **Genes** |
| --- | --- | --- | --- |
| hypersensitive reaction | 14 | 4.5 x10^-11^ | IL8, SELL, SPP1, CXCL9, CCL22, FCGR2B, PLA2G7, PI3, CCL2, CD1A, CORO1A, CCR2, MMP9, PRKCB |
| systemic autoimmune syndrome | 20 | 6.5 x10^-11^ | IL8, SELL, SLC7A11, SPP1, CXCL9, KCNJ15, CCL22, S100A12, CXCR1, CX3CR1, FCGR2B, IL1R2, CCL2, MERTK, CD1A, IDO1, CCR2, MMP9, PRKCB, STEAP4 |
| psoriasis | 14 | 3.1 x10^-9^ | IL8, CXCL9, F13A1, S100A12, LAMP3, PI3, CCL2, HGF, IDO1, CCR2, MMP12, MMP9, ACPP, PRKCB |
| atherosclerotic lesion | 8 | 1.0 x10^-8^ | SELL, SPP1, CCL2, CX3CR1, MMP12, CCR2, MMP9, PRKCB |
| insulin-dependent diabetes mellitus | 12 | 1.4 x10^-8^ | IL1R2, IL8, SELL, SLC7A11, CXCL9, MERTK, CCL2, CCL22, CX3CR1, FCGR2B, CCR2, PRKCB |
| vascular lesion | 9 | 1.5 x10^-8^ | SELL, SPP1, CCL2, CX3CR1, S100A12, MMP12, CCR2, MMP9, PRKCB |
| chronic large plaque psoriasis | 4 | 4.5 x10^-8^ | IL8, CXCL9, CCL2, S100A12 |
| chronic small plaque psoriasis | 4 | 4.5 x10^-8^ | IL8, CXCL9, CCL2, S100A12 |
| rheumatoid arthritis | 14 | 4.5 x10^-8^ | IL8, SPP1, CXCL9, SLC7A11, KCNJ15, S100A12, CXCR1, FCGR2B, IL1R2, CCL2, CD1A, CCR2, MMP9, STEAP4 |
| Arthritis | 16 | 5.8 x10^-8^ | IL8, SPP1, CXCL9, SLC7A11, KCNJ15, S100A12, CXCR1, CLEC5A, FCGR2B, IL1R2, CCL2, CD1A, CCR2, MMP9, PRKCB, STEAP4 |
| diabetes mellitus | 15 | 1.3 x10^-7^ | IL8, SELL, SPINK1, SPP1, CXCL9, SLC7A11, CCL22, CX3CR1, FCGR2B, IL1R2, CCL2, MERTK, HGF, CCR2, PRKCB |
| hydronephrosis | 6 | 1.5 x10^-7^ | IL8, CXCL9, CCL2, HGF, FCGR2B, MMP9 |
| Acne | 6 | 2.4 x10^-7^ | IL1R2, PI3, IL8, SELL, CCR2, MMP9 |
| inflammation of lung | 10 | 2.5 x10^-7^ | IL8, SELL, SPP1, CCL2, CCL22, CX3CR1, MMP12, FCGR2B, CCR2, MMP9 |
| vascular disease | 15 | 2.6 x10^-7^ | IL8, SELL, PLXNC1, SPP1, F13A1, S100A12, CX3CR1, FCGR2B, PLA2G7, CCL2, MERTK, CCR2, MMP12, MMP9, PRKCB |
| leukocytosis | 6 | 3.0 x10^-7^ | IL8, SELL, SPP1, CCL2, CCR2, MMP9 |
| proteinuria | 7 | 3.4 x10^-7^ | SPP1, CCL2, HGF, IDO1, CCL22, FCGR2B, MMP9 |
| glucose metabolism disorder | 16 | 3.7 x10^-7^ | IL8, SELL, SPINK1, SPP1, CXCL9, SLC7A11, CCL22, CX3CR1, FCGR2B, IL1R2, CCL2, MERTK, HGF, CCR2, PRKCB, STEAP4 |
| female genital tract cancer | 14 | 4.0 x10^-7^ | IL8, SPP1, F13A1, LAMP3, PI3, SERPINB9, CCL2, HGF, IDO1, MEF2C, MMP12, MMP9, ETV5, PRKCB |
| Allergy | 10 | 4.7 x10^-7^ | IL8, CXCL9, CD1A, CORO1A, CCL22, FCGR2B, CCR2, PLA2G7, MMP9, PRKCB |
| disorder of artery | 13 | 5.6 x10^-7^ | IL8, SELL, PLXNC1, SPP1, F13A1, S100A12, CX3CR1, PLA2G7, CCL2, CCR2, MMP12, MMP9, PRKCB |
| size of lesion | 8 | 5.6 x10^-7^ | SPP1, HGF, MEF2C, CX3CR1, MMP12, CCR2, MMP9, PRKCB |
| immediate hypersensitivity | 9 | 6.0 x10^-7^ | IL8, CXCL9, CD1A, CORO1A, CCL22, FCGR2B, CCR2, PLA2G7, PRKCB |
| inflammation of liver | 8 | 7.2 x10^-7^ | IL8, SPP1, CCL2, HGF, CCL22, FCGR2B, CCR2, MMP9 |
| plaque psoriasis | 6 | 7.3 x10^-7^ | PI3, IL8, CXCL9, CCL2, S100A12, CCR2 |
