# Supplementary material for: Inflammatory and Repair Pathways Induced in Human Bronchoalveolar Lavage Cells with Ozone Inhalation
Source: PLoS One. 2015 Jun 2;10(6):e0127283. doi: 10.1371/journal.pone.0127283 (PMC4452717; doi:10.1371/journal.pone.0127283)
Supplement: S9 Table — iReport generated 25 diseases that were associated with the 49 DEGs from the two-group comparison of 0 to 200 ppb ozone exposure. Diseases with a p-value <1x10^-6^ are shown. (DOCX) [file pone.0127283.s012.docx]

**S9 Table-**

| **Disease** | **DEGs** | **p-value** | **Genes** |
| --- | --- | --- | --- |
| Hypersensitive reaction | 14 | 4.5 x10^-11^ | IL8, SELL, SPP1, CXCL9, CCL22, FCGR2B, PLA2G7, PI3, CCL2, CD1A, CORO1A, CCR2, MMP9, PRKCB |
| Systemic autoimmune syndrome | 20 | 6.5 x10^-11^ | IL8, SELL, SLC7A11, SPP1, CXCL9, KCNJ15, CCL22, S100A12, CXCR1, CX3CR1, FCGR2B, IL1R2, CCL2, MERTK, CD1A, IDO1, CCR2, MMP9, PRKCB, STEAP4 |
| Psoriasis | 14 | 3.1 x10^-9^ | IL8, CXCL9, F13A1, S100A12, LAMP3, PI3, CCL2, HGF, IDO1, CCR2, MMP12, MMP9, ACPP, PRKCB |
| Atherosclerotic lesion | 8 | 1.0 x10^-8^ | SELL, SPP1, CCL2, CX3CR1, MMP12, CCR2, MMP9, PRKCB |
| Insulin-dependent diabetes mellitus | 12 | 1.4 x10^-8^ | IL1R2, IL8, SELL, SLC7A11, CXCL9, MERTK, CCL2, CCL22, CX3CR1, FCGR2B, CCR2, PRKCB |
| Vascular lesion | 9 | 1.5 x10^-8^ | SELL, SPP1, CCL2, CX3CR1, S100A12, MMP12, CCR2, MMP9, PRKCB |
| Chronic large plaque psoriasis | 4 | 4.5 x10^-8^ | IL8, CXCL9, CCL2, S100A12 |
| Chronic small plaque psoriasis | 4 | 4.5 x10^-8^ | IL8, CXCL9, CCL2, S100A12 |
| Rheumatoid arthritis | 14 | 4.5 x10^-8^ | IL8, SPP1, CXCL9, SLC7A11, KCNJ15, S100A12, CXCR1, FCGR2B, IL1R2, CCL2, CD1A, CCR2, MMP9, STEAP4 |
| Arthritis | 16 | 5.8 x10^-8^ | IL8, SPP1, CXCL9, SLC7A11, KCNJ15, S100A12, CXCR1, CLEC5A, FCGR2B, IL1R2, CCL2, CD1A, CCR2, MMP9, PRKCB, STEAP4 |
| Diabetes mellitus | 15 | 1.3 x10^-7^ | IL8, SELL, SPINK1, SPP1, CXCL9, SLC7A11, CCL22, CX3CR1, FCGR2B, IL1R2, CCL2, MERTK, HGF, CCR2, PRKCB |
| Hydronephrosis | 6 | 1.5 x10^-7^ | IL8, CXCL9, CCL2, HGF, FCGR2B, MMP9 |
| Acne | 6 | 2.4 x10^-7^ | IL1R2, PI3, IL8, SELL, CCR2, MMP9 |
| Inflammation of lung | 10 | 2.5 x10^-7^ | IL8, SELL, SPP1, CCL2, CCL22, CX3CR1, MMP12, FCGR2B, CCR2, MMP9 |
| Vascular disease | 15 | 2.6 x10^-7^ | IL8, SELL, PLXNC1, SPP1, F13A1, S100A12, CX3CR1, FCGR2B, PLA2G7, CCL2, MERTK, CCR2, MMP12, MMP9, PRKCB |
| Leukocytosis | 6 | 3.0 x10^-7^ | IL8, SELL, SPP1, CCL2, CCR2, MMP9 |
| Proteinuria | 7 | 3.4 x10^-7^ | SPP1, CCL2, HGF, IDO1, CCL22, FCGR2B, MMP9 |
| Glucose metabolism disorder | 16 | 3.7 x10^-7^ | IL8, SELL, SPINK1, SPP1, CXCL9, SLC7A11, CCL22, CX3CR1, FCGR2B, IL1R2, CCL2, MERTK, HGF, CCR2, PRKCB, STEAP4 |
| Female genital tract cancer | 14 | 4.0 x10^-7^ | IL8, SPP1, F13A1, LAMP3, PI3, SERPINB9, CCL2, HGF, IDO1, MEF2C, MMP12, MMP9, ETV5, PRKCB |
| Allergy | 10 | 4.7 x10^-7^ | IL8, CXCL9, CD1A, CORO1A, CCL22, FCGR2B, CCR2, PLA2G7, MMP9, PRKCB |
| Disorder of artery | 13 | 5.6 x10^-7^ | IL8, SELL, PLXNC1, SPP1, F13A1, S100A12, CX3CR1, PLA2G7, CCL2, CCR2, MMP12, MMP9, PRKCB |
| Size of lesion | 8 | 5.6 x10^-7^ | SPP1, HGF, MEF2C, CX3CR1, MMP12, CCR2, MMP9, PRKCB |
| Immediate hypersensitivity | 9 | 6.0 x10^-7^ | IL8, CXCL9, CD1A, CORO1A, CCL22, FCGR2B, CCR2, PLA2G7, PRKCB |
| Inflammation of liver | 8 | 7.2 x10^-7^ | IL8, SPP1, CCL2, HGF, CCL22, FCGR2B, CCR2, MMP9 |
| Plaque psoriasis | 6 | 7.3 x10^-7^ | PI3, IL8, CXCL9, CCL2, S100A12, CCR2 |
